# Differential Expression Patterns in Chemosensory and Non-Chemosensory Tissues of Putative Chemosensory Genes Identified by Transcriptome Analysis of Insect Pest the Purple Stem Borer *Sesamia inferens* (Walker)

**DOI:** 10.1371/journal.pone.0069715

**Published:** 2013-07-24

**Authors:** Ya-Nan Zhang, Jun-Yan Jin, Rong Jin, Yi-Han Xia, Jing-Jiang Zhou, Jian-Yu Deng, Shuang-Lin Dong

**Affiliations:** 1 Education Ministry, Key Laboratory of Integrated Management of Crop Diseases and Pests, College of Plant Protection, Nanjing Agricultural University, Nanjing, China; 2 Department of Biological Chemistry and Crop Protection, Rothamsted Research, Harpenden, Hertfordshire, United Kingdom; 3 Department of Plant Protection, Zhejiang Agriculture and Forestry University, Linan, Zhejiang, China; INRA-UPMC, France

## Abstract

**Background:**

A large number of insect chemosensory genes from different gene subfamilies have been identified and annotated, but their functional diversity and complexity are largely unknown. A systemic examination of expression patterns in chemosensory organs could provide important information.

**Methodology/Principal Findings:**

We identified 92 putative chemosensory genes by analysing the transcriptome of the antennae and female sex pheromone gland of the purple stem borer *Sesamia inferens*, among them 87 are novel in this species, including 24 transcripts encoding for odorant binding proteins (OBPs), 24 for chemosensory proteins (CSPs), 2 for sensory neuron membrane proteins (SNMPs), 39 for odorant receptors (ORs) and 3 for ionotropic receptors (IRs). The transcriptome analyses were validated and quantified with a detailed global expression profiling by Reverse Transcription-PCR for all 92 transcripts and by Quantitative Real Time RT-PCR for selected 16 ones. Among the chemosensory gene subfamilies, CSP transcripts are most widely and evenly expressed in different tissues and stages, OBP transcripts showed a clear antenna bias and most of OR transcripts are only detected in adult antennae. Our results also revealed that some OR transcripts, such as the transcripts of SNMP2 and 2 IRs were expressed in non-chemosensory tissues, and some CSP transcripts were antenna-biased expression. Furthermore, no chemosensory transcript is specific to female sex pheromone gland and very few are found in the heads.

**Conclusion:**

Our study revealed that there are a large number of chemosensory genes expressed in *S. inferens*, and some of them displayed unusual expression profile in non-chemosensory tissues. The identification of a large set of putative chemosensory genes of each subfamily from a single insect species, together with their different expression profiles provide further information in understanding the functions of these chemosensory genes in *S. inferens* as well as other insects.

## Introduction

Olfaction plays an important role in various crucial behaviors of insects, such as locating food resources, plant and animal hosts and finding sexual partners. The periphery process of insect olfaction is generally thought to involve two main steps. Firstly, external chemical volatiles enter into the chemosensilla of insect antennae or other sensory tissues, and then are captured by odorant binding proteins (OBPs) [1], [2], [3] or chemosensory proteins (CSPs) [4], [5] which are highly abundant in the lymph of chemosensilla. Secondly, the OBP or CSP bound chemical volatiles are transported to the olfactory receptor proteins (ORs) [6], [7], [8] located on dendrite membranes, triggering the transduction of chemical signals to electric signals. In addition, some other chemosensory proteins have also been proposed to play a role in insect olfaction. Two important ones are sensory neuron membrane proteins (SNMPs) [9], [10] and ionotropic receptors (IRs) [1], [11], [12].

Identification and expression profiling of chemosensory genes are of primary importance for exploring their functions and the mechanisms of insect olfaction. In the early studies, the main method used to identify insect chemosensory genes was direct cloning [13], [14], [15], [16], [17], [18], [19], [20], [21], [22], [23], which normally involves designing degenerate primers, amplifying the fragment and obtaining the full length gene sequences by Rapid Amplification of cDNA Ends (RACE). This method is very time-consuming and inefficient, identifying only one gene each time. Later, the genome sequencing and annotation projects have allowed to find large-scale new chemosensory genes in *B. mori* [24], [25] and several other insect species [25], [26], [27], including first identification of insect ORs from *Drosophila melanogaster* [28]. Recently, with development of the next generation sequencing techniques, large scale chemosensory genes have also been identified from insects whose genomes have not been sequenced, as reported in *Spodoptera littoralis* [29], [30], *Manduca sexta* [31], *Cydia pomonella* [32] and *Helicoverpa armigera* [33].

Although great numbers of chemosensory genes have been molecularly identified from insects of almost all insect orders, their exact functions are mostly unknown, as these genes were identified mainly based on the sequence similarity to reported genes. The expression profiles, particularly the tissue distribution, could provide important information on the functions of the chemosensory genes [24], [34], [35], [36], [37], [38], [39], [40], [41], [42].

The purple stem borer (also called pink stem borer), *Sesamia inferens* (Lepidoptera: Noctuidae) is a polyphagous insect pest found in many Asian countries [43]. It damages a variety of crops including rice, corn, sugarcane, and has become one of the major rice pests in China since 1990s [44], [45]. In this study, we conducted a transcriptome analysis of adult antennae and female sex pheromone glands of *S. inferens*, and identified 92 putative chemosensory transcripts comprising of 24* OBPs*, 24 *CSPs*, 2 *SNMPs*, 39 *ORs* and 3 *IRs*. We further conducted a comprehensive examination on the expression profile of these transcripts regarding to different tissues and life stages by Reverse Transcription-PCR (RT-PCR) for all transcripts and by Quantitative Real Time RT-PCR (qRT-PCR) for selected 16 genes. The results clearly depicted different expression profiles among different chemosensory genes families between chemosensory and non-chemosensory tissues, as well as between adults and larvae developmental stages.

## Results

### Transcriptome Sequencing and Sequence Assembly

**Figure 1:**
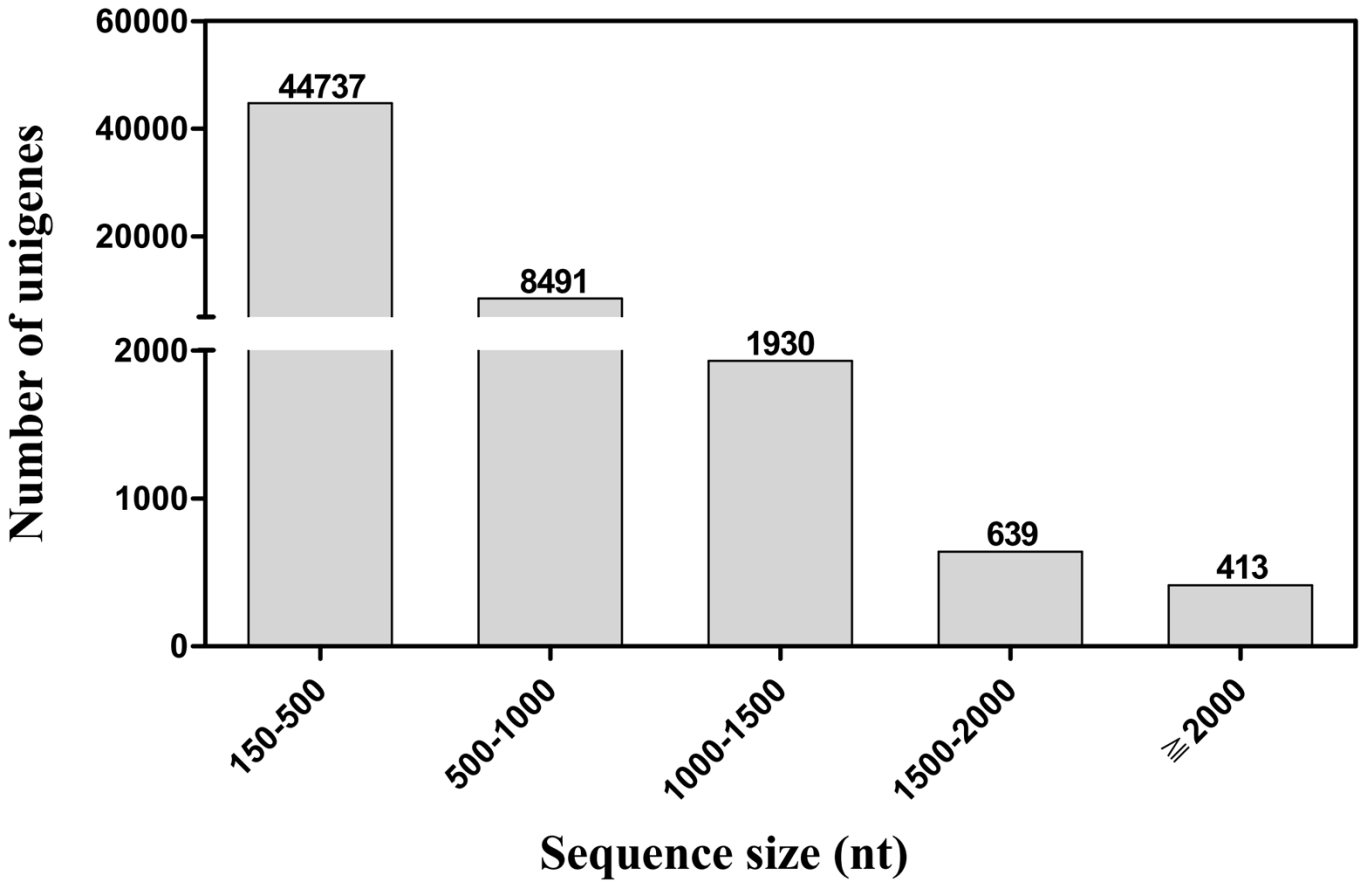
Distribution of unigene size in the *S. inferens* transcriptome assembly.

We carried out a next generation sequencing project on a cDNA library constructed from the mixture sample of antennae and female sex pheromone glands of *S. inferens* using Illuminna HiSeq™ 2000 platform. The transcriptome sequencing provided about 54 million reads (4.86 Gb), which were assembled into 175,059 contigs (≥75 bp) with a mean length of 195 bp and the N50 length of 234 bp. These contigs were further assembled into 126,081 scaffolds with a mean of 243 bp and the N50 length of 308 bp. After clustering and redundancy filtering, we finally acquired 56,210 unigenes (≥150 bp) with a mean length of 394 bp and the N50 length of 460 bp. We called these 56,210 ones unigenses according to some recently published papers [33], [46], although each of them may not necessarily represents a unique gene. Of the 56,210 unigenes, those with a sequence length more than 500 bp accounted for 20.41% of the transcriptome assembly (Figure 1). All the unigenes were referred to as transcripts here after and given a unique unigene id.

### Homology Analysis and Gene Ontology (GO) Annotation

**Figure 2:**
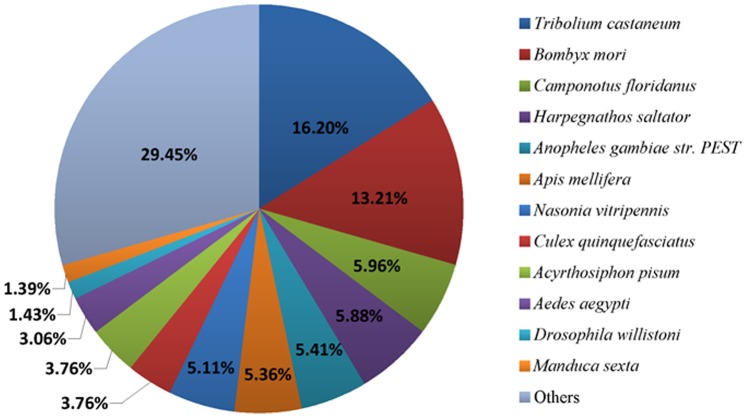
Percentage of homologous hits of the *S. inferens* transcripts to other insect species. The *S. inferens* transcripts were searched by BLASTx against the non-redundancy protein database with a cutoff E-value 10^−5^. Species which have more than 1% matching hits to the *S. inferens* transcripts are shown.

Among 56,210 transcripts, 21,796 were matched by the Blastx homology search to the entries in NCBI non-redundant (nr) protein database with a cut-off E-value of 10^−5^. The highest match percentage (16.20%) is to *Tribolium castaneum* sequences followed by the sequences of *Bombyx mori* (13.21%), *Camponotus floridanus* (5.96%), *Harpegnathos saltator* (5.88%) and *Anopheles gambiae str.* PEST (5.41%) (Figure 2).

**Figure 3:**
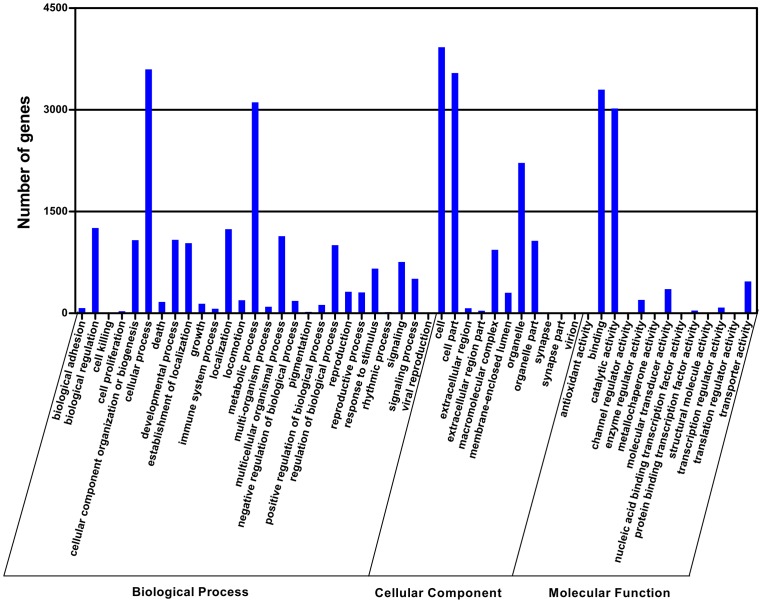
Gene ontology (GO) classification of the *S. inferens* transcripts with Blast2GO program.

**Figure 4:**
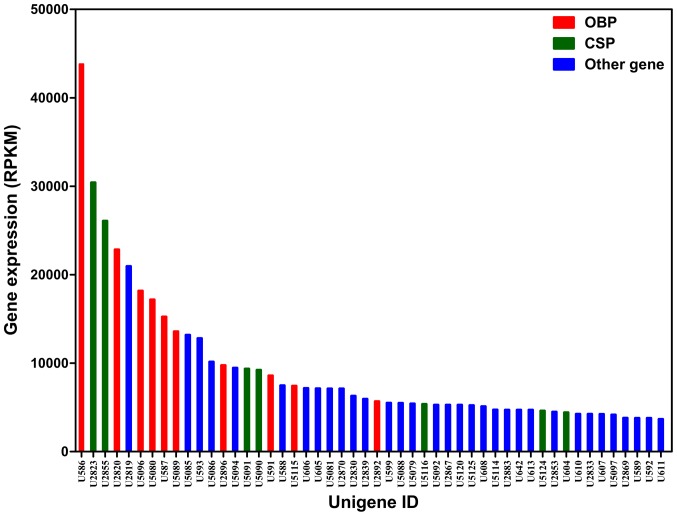
Top 50 most abundant transcripts in the *S. inferens* transcriptome dataset. Odorant binding proteins (PBPs, GOBPs, OBPs and ABP) are indicated by red, chemosensory proteins (CSPs and SAPs) are indicated by green, and the other genes are indicated by blue. The genes expression abundance is indicated as the Reads Per Kilobase per Million mapped reads (RPKM) values. The transcript annotation by homologous comparisons with Blastx is indicated in Table 1 for chemosensory transcripts and Table S1 for the non-chemosensory transcripts.

The Gene Ontology (GO) annotation was used to classify the transcripts into the functional groups according to the GO category. Of 56,210 transcripts, 7,195 ones (12.8%) could be annotated based on sequence homology. As one transcript could align to more than one biological processes, 7,195 transcripts resulted in 18,224 alignments in biological process category, 12119 in cellular component category and 7,509 in molecular function category. In these categories, there were a high percentage of transcripts in the subcategories such as cellular process (49.99%), metabolic process (43.25%), cell (54.54%), cell part (49.25%), binding (45.83%) and catalytic activity (41.97%) (Figure 3). In addition, some chemosensory transcripts were highly abundant in the transcriptome dataset, with 14 of 20 most abundant transcripts encoding for OBPs and CSPs (Figure 4).

### Identification of Putative Chemosensory Genes

**Figure 5:**
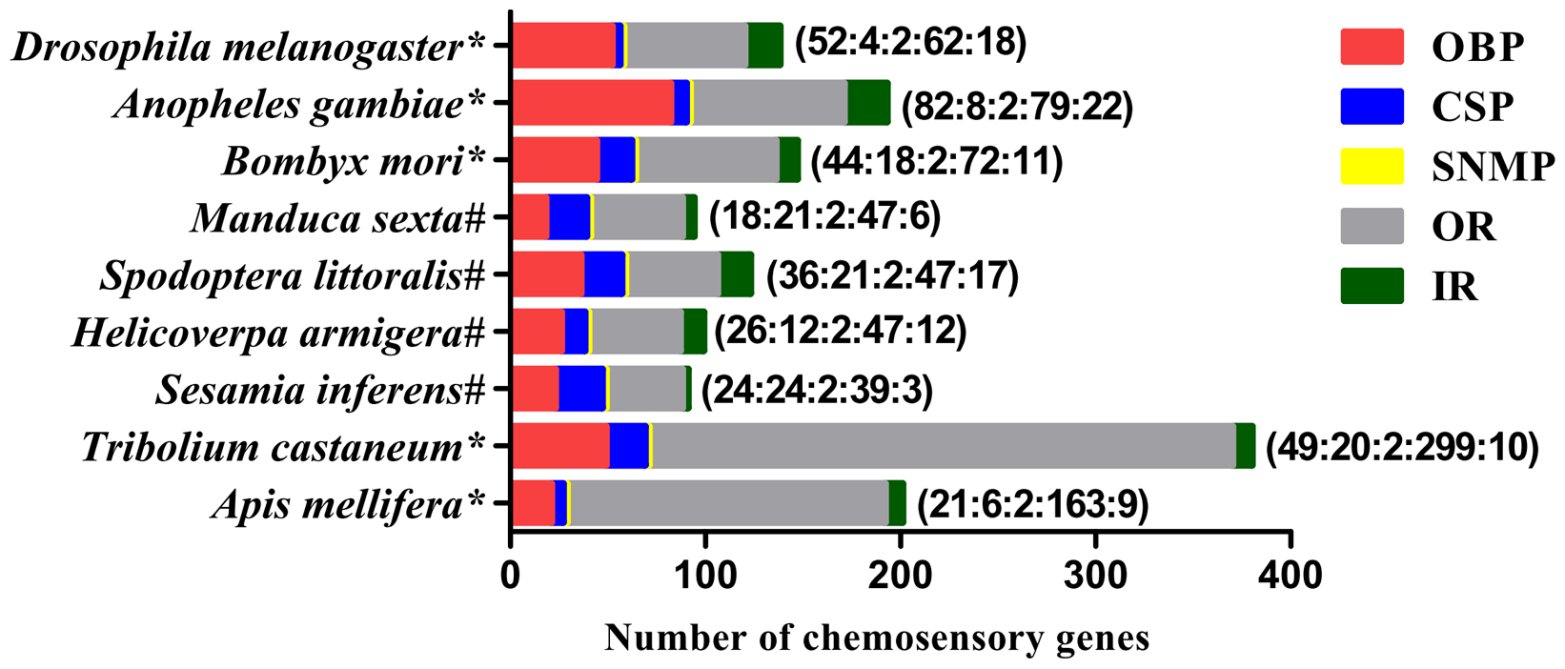
The number of chemosensory genes in different insect species, obtained from genome (*) or antenna transcriptome (#). The digits by the histogram bars represent number of chemosensory genes in different subfamilies (OBP:CSP:SNMP:OR:IR). The data are obtained from the current study for *S. inferens* and from the references [6], [10], [70], [71] for *Drosophila melanogaster*, [6], [10], [70], [71] for *Anopheles gambiae*, [10], [24], [38], [65], [71], [72] for *Bombyx mori*, [6], [10], [70], [71] for *Tribolium castaneum* and *Apis mellifera*, [31] for *Manduca sexta*, [29], [30] for *Spodoptera littoralis* and [33] for *Helicoverpa armigera*.

By homology analysis, we identified a total of 92 transcripts that belong to gene families putatively involved in insect chemoperception, including OBPs (24 transcripts), CSPs (24 transcripts), SNMPs (2 transcripts), ORs (39 transcripts) and IRs (3 transcripts) (Table 1 and Table 2). Of the 92 transcripts, 5 transcripts were the same as sequences deposited in the GenBank: 3 *PBPs* (GenBank accession number: JF927621.1, JN984058.1, JF927622.1), one *GOBP* (EU825760.1) and one *OR* (EU825763.1), while the other 87 transcripts found in the current study were new in *S. inferens*. Compared with insects in which the putative chemosensory genes had been identified by analyzing either genome or transcriptome, the number of the putative chemosensory genes identified by the current study in *S. inferens* (total: 92; OBP : CSP: SNMP : OR: IR = 24∶ 24 : 2∶ 39 : 3) was similar to the numbers found in *M. sexta* (total: 94; OBP : CSP : SNMP: OR : IR = 18∶ 21 : 2∶ 47 : 6) and *H. armigera* (total: 99; OBP : CSP : SNMP : OR : IR = 26∶ 12 : 2∶ 47 : 12), but less than that of *S. littoralis* (total: 127; OBP : CSP : SNMP : OR : IR = 36∶ 21 : 2∶ 47 : 17) and *B. mori* (total: 147; OBP: CSP: SNMP: OR: IR = 44∶18: 2∶72: 11) (Figure 5).

**Table 1:** The Blastx match of *S. inferens* putative *OBPs*, *CSPs* and *SNMPs* genes.

Gene Name	Gene ID	Acc. number	ORF Length (bp)	Complete ORF	Signal Peptide	Best Blastx Match				
						Name	Acc. number	Species	E value	Identity (%)
***Pheromone Binding Protein (PBP)***										
PBP1	2820	AEQ30019.1	312	NO	NO	pheromone binding protein 1	AEQ30019.1	[*Sesamia inferens*]	7.00E-71	99
PBP2	5089	AEX58642.1	177	NO	NO	pheromone binding protein 2	AEX58642.1	[*Sesamia inferens*]	3.00E-33	98
PBP3	620	AEQ30020.1	261	NO	NO	pheromone binding protein 3	AEQ30020.1	[*Sesamia inferens*]	4.00E-57	100
***General odorant binding protein (G0BP)***										
GOBP1	5080	KC887506	495	Yes	Yes	general odorant binding protein 1	ABI24159.1	[*Agrotis segetum*]	3.00E-88	92
GOBP2	586	ACJ07121.1	414	NO	NO	general odorant binding protein 2	ACJ07121.1	[*Sesamia inferens*]	8.00E-96	100
***Odorant Binding Protein (OBP)***										
OBP1	52675	KC887507	321	Yes	NO	odorant-binding protein 3 precursor	NP_001140187.1	[*Bombyx mori*]	2.00E-56	65
OBP2	38179	KC887508	252	NO	NO	odorant binding protein	ACX53743.1	[*Heliothis virescens*]	3.00E-43	82
OBP3	46919	KC887509	345	NO	NO	odorant-binding protein	AEX07274.1	[*Helicoverpa assulta*]	2.00E-72	89
OBP4	34399	KC887510	237	NO	NO	odorant binding protein	ACX53761.1	[*Heliothis virescens*]	4.00E-15	48
OBP5	2896	KC887511	429	NO	Yes	OBP2	B54586.1	[*Helicoverpa armigera*]	2.00E-83	85
OBP6	50001	KC887512	390	Yes	Yes	odorant binding protein LOC100301496 precursor	NP_001153664.1	[*Bombyx mori*]	8.00E-36	41
OBP7	39062	KC887513	237	NO	NO	odorant binding protein	EHJ67765.1	[*Danaus plexippus*]	3.00E-32	68
OBP8	4911	KC887514	303	NO	NO	odorant-binding protein 4	NP_001140188.1	[*Bombyx mori*]	1.00E-39	49
OBP9	43124	KC887515	300	NO	NO	odorant-binding protein 5 precursor	NP_001140189.1	[*Bombyx mori*]	1.00E-36	60
OBP10	5115	KC887516	402	NO	NO	odorant binding protein	ADY17882.1	[*Spodoptera exigua*]	6.00E-74	79
OBP11	24721	KC887517	138	NO	NO	OBP4	AEB54584.1	[*Helicoverpa armigera*]	1.00E-12	57
OBP12	48911	KC887518	348	NO	NO	OBP5	AEB54581.1	[*Helicoverpa armigera*]	1.00E-49	61
OBP13	12194	KC887519	522	Yes	NO	OBP5	AEB54581.1	[*Helicoverpa armigera*]	1.00E-30	48
OBP14	24034		150	NO	NO	antennal binding protein 8	AAL60426.1	[*Manduca sexta*]	3.00E-31	83
OBP15	49422	KC887521	438	NO	NO	antennal binding protein 4	EHJ65654.1	[*Danaus plexippus*]	1.00E-38	70
OBP16	43398	KC887522	222	NO	NO	antennal binding protein 3	AAL60413.1	[*Manduca sexta*]	5.00E-69	84
OBP17	5096	KC887523	213	NO	NO	odorant-binding protein 1	AFG72998.1	[*Cnaphalocrocis medinalis*]	6.00E-34	66
OBP18	19425		169	NO	NO	antennal binding protein 4	AAL66739.1	[*Mamestra brassicae*]	3.00E-32	86
ABPX	587	KC887520	348	NO	NO	antennal binding protein X-1	AAP57463.1	[*Agrotis ipsilon*]	4.00E-55	87
***Chemosensory Protein (CSP)***										
CSP1	18859		150	NO	NO	chemosensory protein 9 precursor	NP_001037069.1	[*Bombyx mori*]	2.00E-26	80
CSP2	21255		183	NO	NO	chemosensory protein	AAF71290.2	[*Mamestra brassicae*]	6.00E-32	89
CSP3	21930		171	NO	NO	chemosensory protein CSP2	ABM67689.1	[*Spodoptera exigua*]	3.00E-25	77
CSP4	27050	KC907741	207	NO	NO	chemosensory protein 8	ADV36661.1	[*Antheraea yamamai*]	2.00E-29	71
CSP5	2822		120	NO	NO	CSP4	AEX07269.1	[*Helicoverpa armigera*]	1.00E-34	89
CSP6	2823	KC907742	387	Yes	Yes	chemosensory protein	AAF71290.2	[*Mamestra brassicae*]	8.00E-75	86
CSP7	2855	KC907743	123	NO	NO	CSP2	AEX07265.1	[*Helicoverpa armigera*]	2.00E-41	91
CSP8	30460	KC907744	219	NO	NO	chemosensory protein	AAF19653.1	[*Mamestra brassicae*]	2.00E-42	83
CSP9	32869	KC907745	203	NO	NO	chemosensory protein 13	BAG71921.1	[*Papilio xuthus*]	1.00E-42	82
CSP10	35445	KC907746	187	NO	NO	chemosensory protein 9 precursor	NP_001037069.1	[*Bombyx mori*]	6.00E-22	73
CSP11	37159	KC907747	207	NO	NO	chemosensory protein	EHJ67380.1	[*Danaus plexippus*]	2.00E-36	87
CSP12	604	KC907748	336	Yes	Yes	sensory appendage protein-like protein	AAK14793.1	[*Mamestra brassicae*]	2.00E-36	65
CSP13	48349	KC907749	375	Yes	Yes	chemosensory protein	ACX53825.1	[*Heliothis virescens*]	9.00E-43	63
CSP14	49098	KC907750	336	Yes	Yes	chemosensory protein	ACX53817.1	[*Heliothis virescens*]	8.00E-49	69
CSP15	50431	KC907751	324	Yes	Yes	chemosensory protein	EHJ67380.1	[*Danaus plexippus*]	8.00E-57	86
CSP16	5090	KC907752	375	Yes	Yes	chemosensory protein	ACX53727.1	[*Heliothis virescens*]	3.00E-46	67
CSP17	5091	KC907753	375	Yes	Yes	chemosensory protein	ACX53727.1	[*Heliothis virescens*]	4.00E-47	66
CSP18	5116	KC907754	435	Yes	Yes	chemosensory protein CSP1	ABM67688.1	[*Spodoptera exigua*]	1.00E-66	76
CSP19	5123	KC907755	369	Yes	Yes	CSP6	AEX07267.1	[*Helicoverpa armigera*]	5.00E-57	86
CSP20	5124	KC907756	387	Yes	Yes	chemosensory protein	AAF71289.1	[*Mamestra brassicae*]	8.00E-71	82
CSP21	591	KC907757	444	Yes	Yes	chemosensory protein CSP1	ABM67686.1	[*Plutella xylostella*]	1.00E-52	57
CSP22	622	KC907758	291	NO	NO	chemosensory protein	ACX53806.1	[*Heliothis virescens*]	3.00E-57	81
CSP23	650	KC907759	384	Yes	Yes	chemosensory protein 2	AAM77040.1	[*Heliothis virescens*]	3.00E-68	83
CSP24	717	KC907760	271	NO	NO	chemosensory protein	ACX53719.1	[*Heliothis virescens*]	4.00E-56	91
***Sensory Neuron Membrane Protein (SNMP)***										
SNMP1	43998	KC907737	270	NO	NO	Sensory neuron membrane protein1	Q8I9S2.1	[*Mamestra brassicae*]	1.00E-69	92
SNMP2	5122	KC907738	1118	NO	NO	Sensory neuron membrane protein2	B2RFN2.1	[*Heliothis virescens*]	0	83

Note: PBP1, PBP2, PBP3 and GOBP2 were previously deposited by others. Genes without accession number represent that the gene fragments obtained in this study were less than 200 bp in length. Gene fragments less than 200 bp are unable to be deposited in the GenBank, and thus no accession numbers were provided for these genes.

**Table 2:** The Blastx match of *S. inferens* putative *ORs* and *IRs* genes.

Gene Name	Gene ID	Acc. number	ORF Length (bp)	Complete ORF	TMD(NO)	Best Blastx Match				
						Name	Acc. number	Species	E value	Identity (%)
***Odorant Receptor (OR)***										
OR1	11700	KC960453	561	NO	4	odorant receptor	AEF32141.1	[*Spodoptera exigua*]	4.00E-94	73
OR2	49820	KC960454	1422	Yes	7	olfactory receptor-2	BAG71415.1	[*Mythimna separata*]	0	96
OR3	11970	KC960455	288	NO	0	olfactory receptor 10	ACC63238.1	[*Helicoverpa armigera*]	2.00E-61	96
OR4	21368		126	NO	0	putative chemosensory receptor 17	CAG38118.1	[*Heliothis virescens*]	9.00E-07	83
OR5	44838	KC960456	264	NO	0	olfactory receptor 12	ACF32963.1	[*Helicoverpa armigera*]	3.00E-61	84
OR6	34021	KC960457	243	NO	0	olfactory receptor 63	NP_001166620.1	[*Bombyx mori*]	3.00E-23	68
OR7	16167		167	NO	0	candidate odorant receptor 2	ACS45308.1	[*Helicoverpa assulta*]	1.00E-24	79
OR8	27099	KC960458	198	NO	0	olfactory receptor-like receptor	BAG12809.1	[*Bombyx mori*]	1.00E-20	60
OR9	52605	KC960459	471	NO	2	olfactory receptor 36	NP_001166892.1	[*Bombyx mori*]	1.00E-65	67
OR10	22505		177	NO	0	putative chemosensory receptor 21	CAG38122.1	[*Heliothis virescens*]	1.00E-15	58
OR11	11122	KC960460	267	NO	1	olfactory receptor 13	NP_001166603.1	[*Bombyx mori*]	2.00E-35	64
OR12	1887	KC960461	354	NO	1	odorant receptor 42	ABK27852.1	[*Bombyx mori*]	2.00E-42	56
OR13	26406	KC960462	192	NO	0	olfactory receptor 60	NP_001155301.1	[*Bombyx mori*]	8.00E-26	75
OR14	34752	KC960463	207	NO	2	putative chemosensory receptor 7	CAD31853.1	[*Heliothis virescens*]	2.00E-24	58
OR15	37297	KC960464	257	NO	1	putative odorant receptor OR12	AFC91721.1	[*Cydia pomonella*]	9.00E-42	76
OR16	39913	KC960465	147	NO	0	putative odorant receptor OR17	AFC91725.1	[*Cydia pomonella*]	2.00E-17	54
OR17	10394	KF008005	246	NO	0	olfactory receptor 33	NP_001103623.1	[*Bombyx mori*]	1.00E-32	57
OR18	10399	KF008006	462	NO	1	olfactory receptor 22	NP_001166613.1	[*Bombyx mori*]	6.00E-87	74
OR19	11474	KC960466	1080	NO	6	olfactory receptor-like	NP_001116817.1	[*Bombyx mori*]	2.00E-162	68
OR20	1458	KC960467	522	NO	3	olfactory receptor 56	NP_001166617.1	[*Bombyx mori*]	6.00E-110	76
OR21	5112	KC960468	1002	NO	2	putative chemosensory receptor 16	CAG38117.1	[*Heliothis virescens*]	9.00E-169	77
OR22	43193	KC960469	300	NO	1	odorant receptor 23	DAA05981.1	[*Bombyx mori*]	1.00E-18	41
OR23	4444	KC960470	732	NO	3	olfactory receptor	EHJ63141.1	[*Danaus plexippus*]	6.00E-77	47
OR24	54083	KC960471	726	NO	3	olfactory receptor 44	NP_001166607.1	[*Bombyx mori*]	2.00E-83	80
OR25	53466	KC960472	647	NO	3	olfactory receptor 49	NP_001166614.1	[*Bombyx mori*]	9.00E-67	59
OR26	53488	KC960473	546	NO	0	odorant receptor 30	DAA05986.1	[*Bombyx mori*]	4.00E-90	68
OR27	53951	KC960474	616	NO	0	olfactory receptor	BAG71423.2	[*Mythimna separata*]	7.00E-114	74
OR28	54580	KC960475	774	NO	4	olfactory receptor 16	NP_001104832.2	[*Bombyx mori*]	5.00E-137	71
OR29	54690	KC960476	624	NO	0	olfactory receptor-1	BAG71414.1	[*Mythimna separata*]	5.00E-143	81
OR30	54930	KC960477	714	NO	3	olfactory receptor 64	NP_001166621.1	[*Bombyx mori*]	4.00E-85	65
OR31	54964	KC960478	750	NO	4	olfactory receptor-like receptor	EHJ72218.1	[*Danaus plexippus*]	8.00E-78	42
OR32	55698	KC960479	1080	NO	5	olfactory receptor 29	NP_001166894.1	[*Bombyx mori*]	6.00E-176	66
OR33	5924	KC960480	672	NO	3	putative odorant receptor OR24	AFC91732.1	[*Cydia pomonella*]	3.00E-83	61
OR34	7341	KF008007	921	NO	4	putative chemosensory receptor 15	CAG38116.1	[*Heliothis virescens*]	1.00E-108	69
OR35	54102	KC960481	435	NO	3	putative chemosensory receptor 21	CAG38122.1	[*Heliothis virescens*]	3.00E-89	75
OR36	55898	KC960482	1209	Yes	6	putative chemosensory receptor 21	CAG38122.1	[*Heliothis virescens*]	1.00E-91	40
OR37	11050	KF008008	336	NO	0	odorant receptor OR24	NP_001155300.1	[*Bombyx mori*]	1.00E-10	44
OR38	2802	KC960483	469	NO	2	olfactory receptor 35	NP_001103476.1	[*Bombyx mori*]	3.00E-36	51
OR39	11752	KC960484	450	NO	2	odorant receptor 38	ABK27851.1	[*Bombyx mori*]	7.00E-49	59
***Ionotropic Receptor (IR)***										
IR93a	11522	KC907739	384	NO	1	ionotropic receptor 93a, isoform B	NP_732567.1	[*Drosophila melanogaster*]	2.00E-23	39
IR75d	14944		168	NO	0	putative chemosensory ionotropic receptor IR75d	ADR64683.1	[*Spodoptera littoralis*]	2.00E-26	95
IR76b	1261	KC907740	1629	Yes	3	putative chemosensory ionotropic receptor IR76b	ADR64687.1	[*Spodoptera littoralis*]	0	84

Note: Genes without accession number represent that the gene fragments obtained in this study were less than 200 bp in length. Gene fragments less than 200 bp are unable to be deposited in the GenBank, and thus no accession numbers were provided for these genes.

Of the 92 chemosensory transcripts, we carried out the validation experiments for the transcripts encoding for 11 OBPs, 3**CSPs, and 6 ORs by RT-PCR and confirmed their identity by sequencing the PCR products. The sequences obtained from positive clones were of ≧99% identical at the nucleic acid level with the corresponding sequences from the transcriptome, indicating that the assembly of the transcripts was adequate.

Among the 87 new putative chemosensory genes, 4 OBPs, 12 CSPs and 3 ORs contained complete open reading frame (ORF); 9 CSPs and one OR (OR2) were of full-length (Table 1 and Table 2). These genes were obtained by transcriptome analysis and RACE.

### Expression Profile of the Putative Chemosensory Transcripts

**Figure 6:**
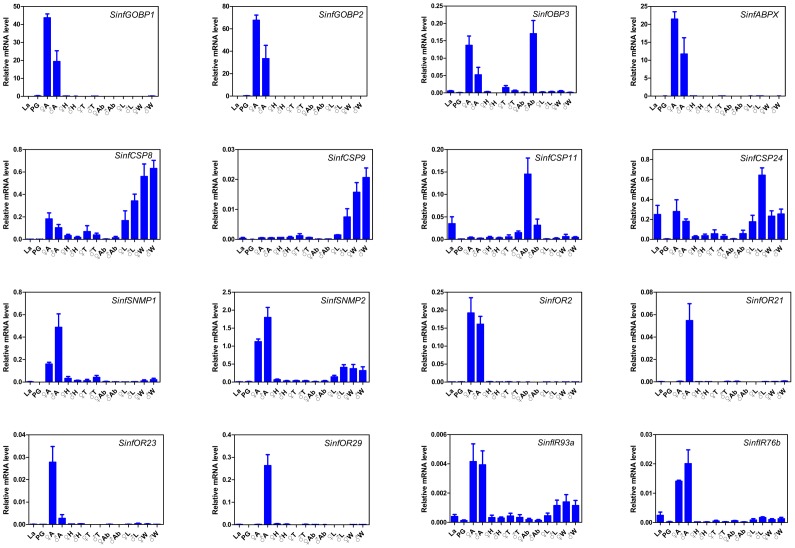
Relative expression levels of 16 putative chemosensory transcripts using qRT-PCR. La, larvae whole body; PG, female pheromone glands; A, antennae; H, heads; T, thoraxes; Ab, abdomens (female without PG); L, legs; W, wings; ♀, female, ♂, male.

To investigate the general expression profiles, RT-PCR measurements for all 92 transcripts were conducted (Table 3, Figure S1 and Table S2), and 16 selected transcripts were further quantified by qRT-PCR (Figure 6) to validate the RT-PCR results. As a result, the overall relative expression profiles of these transcripts in different tissues and stages obtained by the two methods were similar. In addition, there was a clear agreement between transcript abundance estimated by transcritptome analysis and the expression level measured by RT-PCR. Fourteen of top 20 highly abundant transcripts (*Unigene586*, *Unigene2823, Unigene2855, Unigene2820, Unigene5096, Unigene5080, Unigene587, Unigene5089, Unigene2821, Unigene2896, Unigene5091, Unigene5090, Unigene591* and *Unigene5115*) (Figure 4) were highly expressed in the antennae (*GOBP2*, *CSP6, CSP7, PBP1, OBP16, GOBP1, ABPX, PBP2, PBP3, OBP5, CSP17, CSP16, CSP21* and *OBP10*) (Table 3). This suggested that the RT-PCR could be used as an effective mean to investigate the general expression profiles and the relative levels of the putative chemosensory genes among different tissues and developmental stages.

**Table 3:** Expression of putative chemosensory genes in larvae and different adult tissues of *S. inferens*.

Gene	Tissue													
	La	PG	A♀	A♂	H♀	H♂	T♀	T♂	Ab♀	Ab♂	L♀	L♂	W♀	W♂
***Pheromone Binding Protein (PBP)***														
PBP1	*		***	***			*	*	*	*		*		
PBP2			***	***			*	*	*	*	**	**	*	*
PBP3	*		***	***			*		*	*	*	*	*	*
***General odorant binding protein (GOBP)***														
GOBP1		*	***	***			*	*	*	*	*	*	*	*
GOBP2			***	***					*	*	*	*		
***Oorant binding protein (OBP)***														
OBP1^a^			***	**										
OBP2^a^	**	**	*	*			**	*	***	***	**	**	**	**
OBP3	*		***	***			*	*	*	***	*	*		
OBP4	***	**	**	**			**	**	**	**	**	**	**	**
OBP5			***	***			*	*			*	**	*	
OBP6	*		***	***							*	*		
OBP7^a^	**		**	**					*					
OBP8			***	***							*	*		
OBP9	*	*	*	*							*	*	*	*
OBP10^a^		*	***	***			*	*		*	*	*		
OBP11^ a^		*	***	***				**		*	***	**	**	**
OBP12	*		**	*							**	*		
OBP13	**	**	***	**				*	*		*	**	**	*
OBP14	***	**	***	***	*	*	***	***	**	**	***	***	**	**
OBP15^a^			***	**							*			
OBP16^a^			***	***							*	*		
OBP17			***	***							*	*		
OBP18	*	***	***	***			***	***	***	***	***	***	***	***
ABPX^a^			***	***							*	*		
***Chemosensory Protein (CSP)***														
CSP1	**	***	***	***			***	***	**	**	***	***	***	***
CSP2	**	**	**	**	*	*	***	**	**	**	***	***	***	***
CSP3	**	**	***	***		**	**	***	**	**	**	***	***	***
CSP4	***	***	***	***			**	**	*	**	***	***	***	***
CSP5	***	**	***	***	*	*	**	***	*	**	***	***	***	***
CSP6	***	***	***	***	*	*	***	***	***	***	***	***	***	***
CSP7	*	***	***	***	*	*	***	***	***	***	***	***	***	***
CSP8^a^	*	*	***	***			**	**	*	**	***	***	***	***
CSP9^a^			*	*							*	**	**	**
CSP10	**	*	***	***			***	***	*	**	**	***	***	***
CSP11^a^	**	*	**	*				*	***	**				
CSP12		*	***	***			*		**	*	***	***	***	***
CSP13^a^	***	***	***	***			**	**	**	**	***	***	***	***
CSP14^a^	**	**	**	**				**	**	**	***	***	**	**
CSP15	***	***	***	***			***	***	***	***	***	***	***	***
CSP16	***	***	***	***	*	*	***	***	***	***	***	***	***	***
CSP17	**	**	***	***			***	***	**	*	**	***	***	***
CSP18	***	***	***	***			***	**	**	**	***	***	***	***
CSP19^a^		*	***	***			**	*	*	*	**	**	**	**
CSP20	***	**	***	***	*	*	***	***	**	*	***	***	***	***
CSP21^a^		*	***	***			*	*		*	**	**	*	*
CSP22^a^	***	**	***	***			***	***	***	***	***	***	***	***
CSP23	***	***	***	***	*	*	***	***	***	***	***	***	***	***
CSP24	***	*	***	***			**	**	**	**	***	***	***	***
***Sensory Neuron Membrane Protein (SNMP)***														
SNMP1			***	***	*				*	*	*		*	
SNMP2	*	***	***	***	?	?	**	**	**	**	**	***	***	***
***Odorant Receptor (OR)***														
OR1			***	***										
OR2(OR83b)^a^			***	***										
OR3^a^			*	*										
OR4^a^			**	**										
OR5^a^			***	**										
OR6^a^		**	**	**										
OR7				***										
OR8			**	**										
OR9			***	***										
OR10			**	**										
OR11			***	***										
OR12			***	***										
OR13			***	***										
OR14			**	**										
OR15			***	***										
OR16^a^			***											
OR17			**	**										
OR18			*	*										
OR19^a^			***	***										
OR20			***	**										
OR21^a^			*	***										
OR22^a^			*	*										
OR23			***	*										
OR24			***	**										
OR25	*	**	***	***	*		**	**	***	**	**	**	**	**
OR26^a^			***	**										
OR27^a^			**	***										
OR28			*	*										
OR29				***										
OR30			***	***										
OR31^a^			***	***										
OR32	*	**	***	***					**	*	*			
OR33^a^	**	*	***	***				*		*		*		*
OR34	**	*	***	***	*	*			**	*	**	*	*	*
OR35			**	**										
OR36^a^			**	**										
OR37^a^			**	*										
OR38^a^			***	**										
OR39	*		**	**	*				*	*	*		*	
***Ionotropic Receptor (IR)***														
IR93a	*	*	**	**						*		*		*
IR75d		*	**	**			*		*					
IR76b	**		**	**										

The relative expression levels of genes in the same tissue were calculated by the ratio of the RT-PCR bands intensity between target gene and internal reference gene *SinfGAPDH* [73](Figure S1). *, **and *** indicate the intensity ratio of 0.20-0.59, 0.60-0.99, 1.00-1.39, respectively; the blank indicates no signal. The band intensity was calculated by Bio-Rad-Quantity one 4.6.2 software). La, larvae (third instar); Adult tissues include PG, pheromone glands; A, antennae; H, heads (without antennae); T, thoraxes; Ab, abdomens (female without PG); L, legs and W, wings. ♀: female, ♂: male. Superscript “a” followed the gene name represents that the expression level of the gene was obtained by two biological replications.

**Figure 7:**
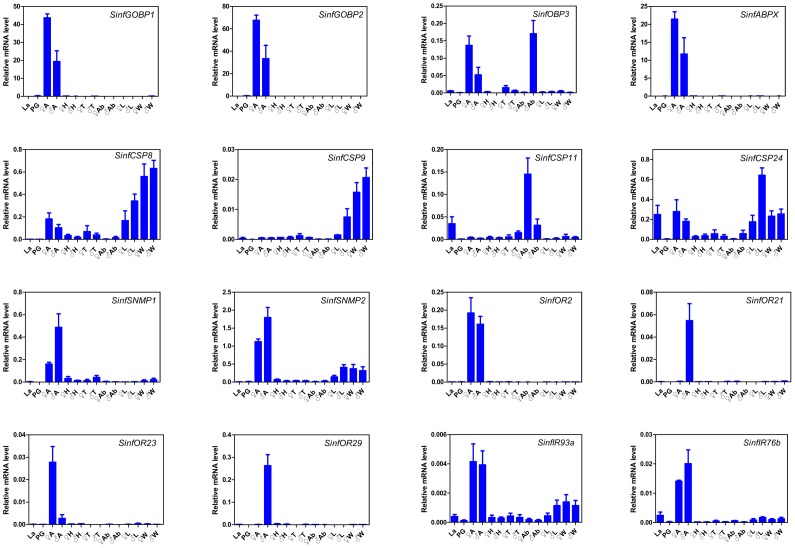
Tissue distribution of the 92 *S. inferens* chemosensory transcripts. A: The proportion of chemosensory genes expressed in larvae, female pheromone gland and other tissues of male and female adults. B: The number of chemosensory gene in each subfamily expressed in larvae, female pheromone glands, and female and male antennae. The digits by the histogram represent number of genes in each subfamily (OBP:CSP:SNMP:OR:IR).

The investigation showed that almost all the transcripts were expressed in the antennae, 40–50% expressed in other tested tissues and only <15% expressed in heads. In addition, the numbers of detected transcripts were similar in male and female moth antennae (91 and 90, respectively), showing no sex bias in chemosensory gene expression (Figure 7A). Thirty nine chemosensory transcripts were detected in female pheromone glands and larvae (Figure 7B).

### OBP Transcript Expression

The tissue expression profiles are shown in Table 3 and Figure 6. Interestingly, *OBP1* was the only antenna-specific OBP transcript. The 3 PBP transcripts and 2 GOBP transcripts displayed highly antenna biased expression, and other antenna highly expressed transcripts included *OBP5, OBP6, OBP8, OBP10, OBP15, OBP16, OBP17* and *ABPX*. The transcripts *OBP4* and *OBP18* had a similar expression level between antennae and non-antenna tissues. *OBP14* was the only OBP transcripts found in all tissues.

Interestingly, the transcripts of *PBP1, PBP3* and others (*OBP2*, *OBP3*, *OBP4*, *OBP6*, *OBP7*, *OBP9*, *OBP12*, *OBP13*, and *OBP14*) were also detected in the larvae. Three PBP transcripts were not detected in the pheromone glands, while *GOBP1*, *OBP*2, *OBP4, OBP9*, *OBP10*, *OBP11*, *OBP13* and *OBP14* were detected in the pheromone glands (Table 3, Figure 6 and Figure S1).

### CSP Transcript Expression

Compared to OBP transcripts, CSP transcripts were highly expressed in non-olfactory tissues as well as olfactory tissues. Among the 24 newly identified CSP transcripts, 21 displayed a wide range of tissue distribution, and 7 CSP transcripts (*CSP2, CSP5-7, CSP16, CSP20* and *CSP23*) were expressed in all 14 tissues. Most of CSP transcript*s* were highly expressed in larvae and in pheromone glands (Table 3, Figure 6 and Figure S1).

### SNMP Transcript Expression

Two *SNMPs* homologs were also obtained from *S. inferens* transcriptome. In comparison, *SNMP1* encoding a protein with 78% identity to SNMP1 of *B. mori* (GenBank accession number: NP_001037186) was highly expressed in the antennae, whilst *SNMP2* encoding a protein with 83% identity to SNMP2 of *Heliothis virescens* (GenBank accession number: B2RFN2.1) was also expressed in remarkable levels in other tissues such as legs and wings (Table 3, Figure 6 and Figure S1).

### OR Transcript Expression

Of the 39**OR transcripts identified in *S. inferens*, 34 were expressed only in antennae of both sexes at lower level, relative to the expression level of the OBP and CSP transcripts. *OR16* was female-specific while *OR7* and *OR29* were male-specific. In addition, two *ORs*, *OR23* and *OR26* were expressed at much higher levels in female antennae than in male antennae, while *OR27* and *OR21* were more highly expressed in male antennae than in female antennae. Only 5 OR transcripts, (*OR6*, *OR25*, and *OR32-34*) were expressed broadly in several tissues, including the female sex pheromone glands and the larvae (Table 3, Figure 6 and Figure S1).

### IR Transcript Expression

All 3 IR transcripts of *S. inferens* were expressed at a high level in the antennae, and also at low levels in other tissues. In comparison, *IR76b* was more specifically detected in the antennae than the other two *IRs* (Table 3, Figure 6 and Figure S1).

### Phylogenetic Analyses

**Figure 8:**
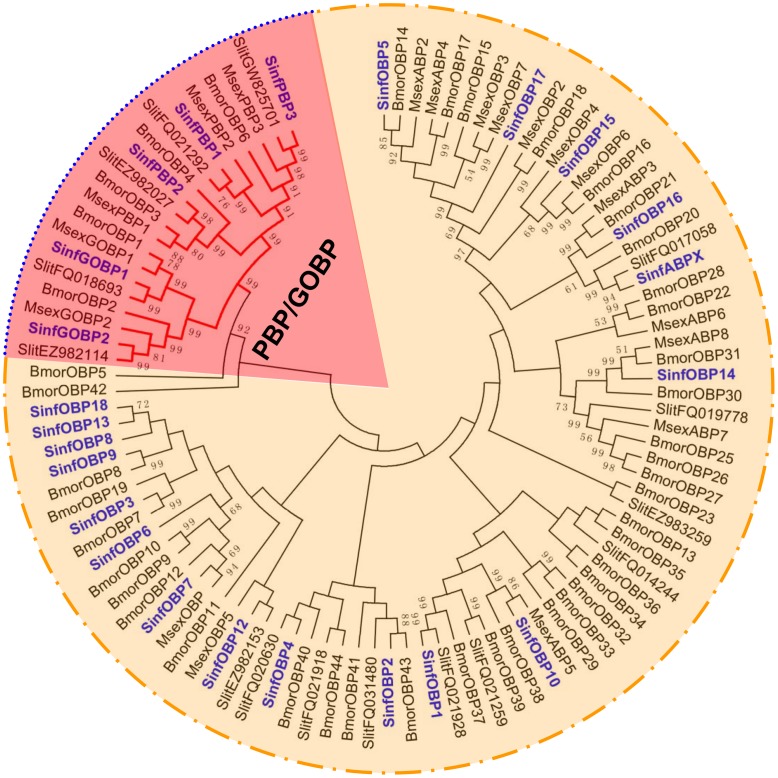
Phylogenetic tree of putative OBPs from *S. inferens*, *M. sexta*, *S. littoralis* and *B. mori*. PBP/GOBP clade is marked in red. The *S. inferens* translated unigenes are shown in blue. Accession numbers are given in Table S3. The tree was constructed with MEGA5.0, using the neighbour-joining method. Values indicated at the nodes are bootstrap values based on 1000 replicates, and the bootstrap values <50% are not shown. Sinf, *Sesamia inferens*; Msex, *Manduca sexta*; Slit, *Spodoptera littoralis*; Bmor, *Bombyx mori*.

**Figure 9:**
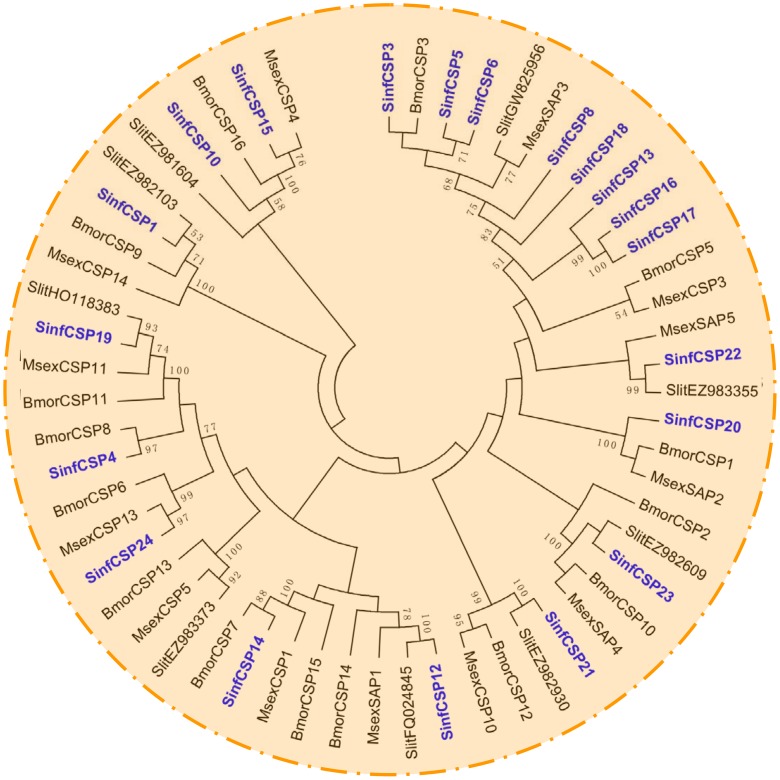
Phylogenetic tree of putative CSPs from *S. inferens*, *M. sexta*, *S. littoralis* and *B. mori*. The *S. inferens* translated unigenes are shown in blue. Accession numbers are given in Table S3. The tree was constructed with MEGA5.0, using the neighbour-joining method. Values indicated at the nodes are bootstrap values based on 1000 replicates, and the bootstrap values <50% are not shown. Sinf, *Sesamia inferens*; Msex, *Manduca sexta*; Slit, *Spodoptera littoralis*; Bmor, *Bombyx mori*.

A phylogenetic tree of OBPs was constructed using protein sequences of the OBPs from *S. inferens*, *M. sexta*, *S. littoralis* and *B. mori* (Figure 8). It was shown that all PBP and GOBP sequences were clustered into distinct clades from other OBPs. More interestingly, the identified SinOBP sequences were clustered in each subclass (PBP1, PBP2, PBP3, GOBP1 and GOBP2) with at least one lepidopteran orthologue (Figure 8). Among the 24 putative CSPs, 20 sequences were clustered with at least one lepidopteran orthologous gene (Figure 9).

**Figure 10:**
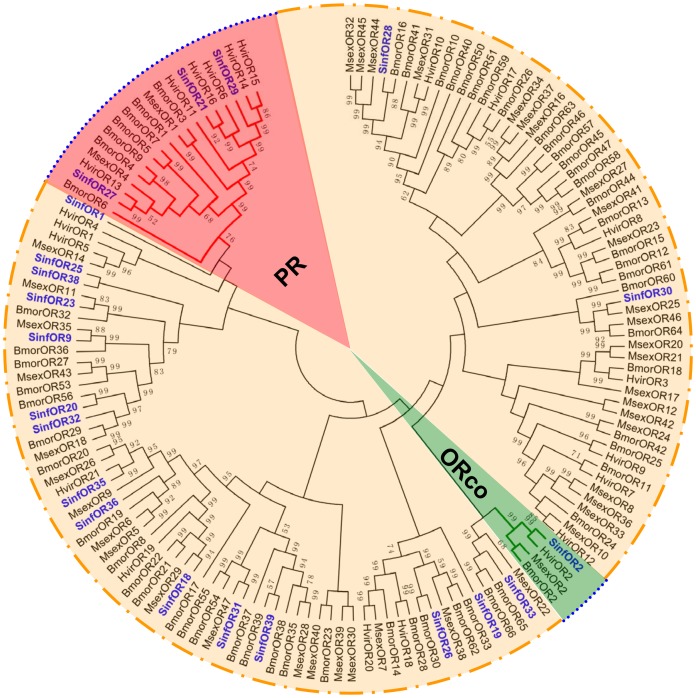
Phylogenetic tree of putative ORs from *S. inferens*, *M. sexta*, *H. virescens* and *B. mori*. PR clade is marked in red and ORco in green. The *S. inferens* translated unigenes are shown in blue. Accession numbers are given in Table S3. The tree was constructed with MEGA5.0, using the neighbour-joining method. Values indicated at the nodes are bootstrap values based on 1000 replicates, and the bootstrap values <50% are not shown. Sinf, *Sesamia inferens*; Msex, *Manduca sexta*; Hvir, *Heliothis virescenss*; Bmor, *Bombyx mori*.

In the OR phylogenetic tree, *SinfOR2* was clustered with other lepidopteran OR2 (ORco) sequences, and three SinfORs (*OR21*, *OR27* and *OR29*) were clustered in the lepidopteran pheromone receptor (PR) clade (Figure 10). The majority of the identified SinfORs had at least one lepidopteran orthologue, with only two (*SinfOR1* and *SinfOR19*) having no counterpart.

## Discussion

In the *S. inferens* transcriptome data of this study, only 38.8% of 56,210 transcripts have homologous matches to the entries of GenBank with the cutoff value of 10^−5^, and only 12.8% can be annotated to one or more GO term by the GO analyses, which is similar to *M. sexta* [31] and *S. littoralis* [30], indicating that a large number of *S. inferens* transcripts are either non-coding or homologous with genes that do not have any GO term. In addition, 87 chemosensory transcripts are first reported in *S. inferens*. Further studies using this transcriptome data could provide insights into insect physiology and pest control strategy [47].

The total number (92) of chemosensory transcripts identified in the current study is similar with those reported in *M. sexta* (94) and *H. armigera* (99), but much lower than those of 5 species whose genome has been sequenced, *D. melanogaster*, *A. gambiae*, *B. mori*, *T. castaneum* and *A. mellifera*. The chemosensory gene numbers in *B. mori* (147) and *S. littorallis* (127) is 1.6 and 1.4 times, respectively of that in *S. inferens* (Figure 5), suggesting there is a high chance to identify more *S. inferens* chemosensory genes. On the other hand, CSP transcripts found in *S. inferens* (24) are more than the CSP genes identified in *B. mori* genome (18) and in *D. melanogaster* genome (4). Therefore, it is more likely that we have identified all the CSPs, while missed out some larvae-biased OBPs and lower expressed ORs. These also imply the plant host adaptation and species-specific sex pheromone perception of lepidopteran insects during evolution.

The phylogenetic analysis of SinfOBPs, SinfCSPs and SinfORs suggest that the identified chemosensory transcripts in *S. inferens* covered main repertoires of the chemosensory genes of the insect. It is worth noting that two ORs (*SinfOR1* and *SinfOR19*) had no counterpart in other species, indicating that the two ORs may represent new types of OR. However, as *SinfOR1* was a fragment with only 187 amino acids, it is possible that counterparts might be found, when the full length sequence is available and used in the analysis.

The tissue distribution profiles of all 92 *S. inferens* chemosensory genes were investigated by RT-PCR, which were confirmed by an additional qRT-PCR measurementusing16 selected genes. Among three subfamilies (CSPs, OBPs and ORs) of the chemosensory gene, *CSPs* are highly expressed and most widely distributed in chemosensory tissues as well as in non-chemosensory tissues, suggesting CSPs in insects may also involve in other functions apart from chemosensation [48], [49], [50], such as female survival and reproduction in *Spodoptera exigua* [51], limb regeneration in *Periplaneta americana* [52] and embryo development in *Apis mellifera* [53]. In our present study, *OBPs* are usually highly expressed in the antennae relative to other chemosensory tissues (legs, wings, female sex pheromone glands). However, about half the OBP transcripts are also weakly expressed in non-chemosensory tissues (thorax and abdomen) (Figure 7A), indicating that these OBPs may also have other functions. On the other hand, OBP transcripts that are exclusively expressed in antennae and legs (such as *PBPs*, *GOBPs*, *OBP8*, *OBP15-17* and *ABPX*) may play important role in chemosensory. Interestingly, both *PBP1* and *PBP3* were detected with weak signals in larvae, similar to that reported in *S. littoralis* larvae [54]. Poivet et al (2012) suggested that the *S*. *littoralis* PBPs in larvae were used to perceive the sex pheromone adsorbed on or deposited on the eggs when female moths ovipositing on the leaves of the host plants, and this perception thus could promote the food search. The larva-expressed PBPs may play similar roles in *S. inferens*.

In contrast to *CSPs* and *OBPs*, *OR* transcripts are highly restricted in the antennae and expressed at lower levels. This olfactory tissue specific expression profile is well consistent with the specific functional role that OR gene family plays in the moth olfaction [7], [55], [56], [57]. Our study also revealed some OR transcripts (*OR25*, *OR33* and *OR34*) have a very high expression level in non-chemosensory tissues (thoraxes and abdomens). It is interesting that two SNMP transcripts displayed very different expression profiles, with SNMP1 being highly antennal biased, while *SNMP2* was ubiquitously expressed in most tested tissues and larvae. This may suggest that SNMP1 is important in chemosensory, while SNMP2 have other functions in addition to (if any) chemosensation.

In conclusion, we identified members within each subfamily of chemosensory gene family by analysing the trancriptomic sequencing data of antennae and female sex pheromone glands from *S. inferens*. This provides a rich resource for investigation and elucidation of the chemosensation in *S. inferens*. As the first step towards understanding their functions, we conducted a comprehensive and comparative examination of the chemosensory gene expression patterns, and demonstrated a wide distribution of these chemosensory proteins. In particular, the expression of SNMPs, IRs and some ORs in non-chemosensory tissues indicate new insights on their roles in insect physiology.

## Materials and Methods

### Insects Rearing and Collection

The purple stem borer *S. inferens* was originally collected from a rice field in the Jiangsu Provincial Academy of Agricultural Sciences, Nanjing, China. To collect the insect naturally occurred in the above mentioned field, ethical approval was not required, because the purple stem borer is a common insect pest in South China including Nanjing city, and the insects in the above mentioned field was naturally occurred without any special property. The collected larvae were reared on fresh wild rice stem in glass bottles (d = 7cm, h = 11cm) until pupation and sexed as pupae [58]. Rearing conditions were 28±1°C, 70–80% RH and a 14 h light:10 h dark photoperiod. Adults were provided with a cotton swab dipped in 10% honey solution and renewed daily. Antennae of both sexes and female pheromone glands of 1–5 day-old adults were collected for transcriptome sequencing. Antennae from 3-day-old adults of both sexes were collected for PCR validation of the chemosensory gene sequences obtained from transcriptomic analysis. Antennae, heads (without antennae), thoraxes, abdomens (female without pheromone glands), legs and wings from 3-day-old virginal male and female, female sex pheromone glands of same adult age, and larvae of third instar were dissected and collected in two replications for detection of the tissue expression by RT-PCR. All samples were collected during the first hour of the photoperiod and stored at -70°C until use.

### cDNA Library Construction and Illumina Sequencing

Total RNA was extracted using TRIzol reagent (Invitrogen), cDNA library construction and Illumina sequencing of the sample were performed at Beijing Genomics Institute (BGI)-Shenzhen, Shenzhen, China [59]. The mRNA was purified from 20 µg of total RNA (a mixture of RNAs from antennae and pheromone glands at 5∶1 ratio) using oligo (dT) magnetic beads and fragmented into short sequences in the presence of divalent cations at 94°C for 5 min. Then, the first-strand cDNA was generated using random hexamer-primed reverse transcription, followed by synthesis of the second-strand cDNA using RNaseH and DNA polymerase I. After the end repair and ligation of adaptors, the products were amplified by PCR and purified using the QIAquick PCR Purification Kit to create a cDNA library, and sequenced on the HisSeq™ 2000 platform.

### *De novo* Assembly of Short Reads and Gene Annotation

Transcriptome de novo assembly is carried out with short reads assembling program SOAPdenovo [60]. SOAPdenovo first combines reads with a certain length of overlap, to form longer fragments without N (N represent unknown sequence) to produce contigs. The reads are then mapped back to contigs, by using paired-end reads that enable identification of contigs from the same transcript and the distances between these contigs. Next, SOAPdenovo connects the contigs based on the paired-end reads for gap filling between each two contigs to build scaffold sequences with the least Ns. Such sequences are defined as unigenes. In this study, all the clean reads were submitted and available from the NCBI/SRA data base (SRA experiment accession number: SRX286371, BioProject accession number: PRJNA205103).

The Unigenes larger than 150 bp were first aligned by BlASTX to protein databases, including Nr, Swiss-Prot, KEGG and COG (e-value<10^−5^), retrieving proteins with the highest sequence similarity with the given unigenes along with their protein functional annotations. Then, we used Blast2GO program [61] to get GO annotation of the unigenes, and got GO functional classification by using WEGO software [62].

### Expression Abundance Analysis of the Unigenes

The expression abundance of these unigenes were calculated by the RPKM (Reads Per Kilobase per Million mapped reads) method [63], using the formula: RPKM (A) = (10,00,000×C×1,000)/(N×L). In the formula, RPKM (A) is the expression abundance of gene A; C is the number of reads that uniquely aligned to gene A; N is total number of reads that uniquely aligned to all genes; and L is the number of bases on gene A. The RPKM method is able to eliminate the influence of different gene lengths and sequencing discrepancy on the calculation of expression abundance.

### RNA Isolation and cDNA Synthesis for Reverse Transcription-PCR

Total RNA was extracted by SV 96 Total RNA Isolation System (Promega, Madison, WI, USA) following the manufacturer’s instructions, in which a DNase digestion was included to avoid the genomic DNA contamination. RNA quality was checked with a spectrophotometer (NanoDropTM 1000, Thermo Fisher Scientific, USA). The single-stranded cDNA templates were synthesized using 1.2 µg total RNAs from various samples with oligo (dT) 18 primer as the anchor primers. The M-MLV Reverse Transcriptase (M-MLV) (TaKaRa, Dalian, Liaoning, China) was used for the cDNA synthesis, with the reaction conducted at 42°C for 1 h, and then stopped by heating at 70°C for 15 min.

### RACE Amplification and Sequence Analysis

The SMART™ RACE cDNA Amplification Kit (Clontech, Mountain View, CA, USA) was used to amplify the 5′ and 3′ regions of target genes following the manufacturer’s instructions. The RACE PCR products were subcloned into pEASY-T3 cloning vector system (TransGene, Beijing, China) and positive clones were sequenced by GenScript (Nanjing, China). Full-length sequences were determined by assembling the cDNA fragments and the sequences obtained from the 5′ and 3′ RACE PCR. The RACE primers (Table S4) were designed using Primer Premier 5.0 (PREMIER Biosoft International, CA, USA).

The open reading frames (ORFs) of the putative chemosensory genes were predicted by using ORF finder (http://www.ncbi.nlm.nih.gov/gorf/gorf.html). The similarity searches were performed by using the NCBI-BLAST network server (http://blast.ncbi.nlm.nih.gov/). Putative N-terminal signal peptides of SinfOBPs and SinfCSPs were predicted by Signal IP 4.0 (http://www.cbs.dtu.dk/services/SignalP/) [64]. The TMDs (Transmembrane Domain) of SinfORs and SinfIRs were predicted using TMHMM Server Version2.0 (http://www.cbs.dtu.dk/services/TMHMM).

### Phylogenetic Analyses

The phylogenetic trees were reconstructed for phylogenetic analyses of SinfOBPs, SinfCSPs and SinfORs, based on the amino sequences (the signal peptides of sequences had been removed) of the putative chemosensory genes and the sequences of other Lepidoptera insects. The OBP data set contained 23 sequences from *S. inferens* (amino acids >45 aa), 19 from *M. sexta* [29], [31] and 43 from *B. mori*. The CSP data set contained the 20 sequences from *S. inferens* (amino acids >40 aa), 13 from *M. sexta* [31], 9**from *S. littoralis* [29] and 15 from *B. mori* [26]. The OR data set contained 21 OR sequences from *S. inferens* (amino acids >144 aa), 43 from *M. sexta* [31], 21 from *H. virescens* [41], [42] and 60 from *B. mori* [65]. The protein name and accession number of the genes used for phylogenetic tree building are listed in Table S3. Amino acid sequences were aligned with ClustalX 2.0 [66] and unrooted trees were constructed by MEGA5.0 [67] using the Neighbor-joining method, with Poisson correction of distances. Node support was assessed using a bootstrap procedure base on 1000 replicates.

### Reverse Transcription-PCR Analysis

Gene specific primers across ORF of predicted chemosensory genes were designed using Beacon Designer 7.6 and Primer Premier 5.0 (PREMIER Biosoft International, CA, USA). The sequences of these primers were listed in Table S4. PCR experiments including negative controls (no cDNA template) were carried out in a MyCycler™ (Bio-Rad, USA) under the following conditions: 94°C for 4 min; 30 (35 for OBP13) cycles at 94°C for 30 sec, 60°C for 30 sec, and 72°C for 40 sec, and final incubation for 10 min at 72°C. The reactions were performed in 12.5 µl with 0.5 µl of single-stranded cDNA, 2.0 mM MgCl_2_, 0.2 mM dNTP, 0.4 µM for each primer and 1.25 U rTaq DNA polymerase (TaKaRa, Dalian, Liaoning, China). PCR products were analyzed by electrophoresis on 2.0% w/v agrose gel in TAE buffer (40 mmol/L Tris-acetate, 2 mmol/L Na_2_EDTA·H_2_O) and the resulting bands were visualized with ethidium bromide and digitized using a GelDoc 2000 (Bio-Rad, USA). The control gene encoding for the *S. inferens* glyceraldehyde-3-phosphate dehydrogenase (*SinfGAPDH*) was used for quantification.

To detect the relative expression levels of the predicted chemosensory genes, the gels loaded with PCR products of different tissues were scanned for quantification of the band intensity, by using Bio-Rad-Quantity one 4.6.2 software. In addition, 32 transcripts were randomly chosen to perform a second biological replication in order to check the repeatability of the tissue expression. To validate the predicted sequences of chemosensory genes, the PCR products obtained from cDNA sample of adult antennae were purified using the AxyPrep™ PCR Cleanup Kit (Axygen), and then sub-cloned into a T/A plasmid using the p*EASY*-T3 cloning vector system (TransGene, China) following manufacturer's instructions. The plasmid DNA was used to transform into Trans1-T1 competent cells. Positive clones were checked by PCR and were sequenced by GenScript (Nanjing, China).

### Quantitative Real Time-PCR Validation

The expression profiling of a total of 16 putative chemosensory genes was carried out to validate the accuracy of the RT-PCR results using quantitative real time-PCR (qRT-PCR) experiments. The qRT-PCR was performed on an ABI 7500 (Applied Biosystems, Foster City, CA, USA) using a mixture of 10 µl 2× SYBR Green PCR Master Mix, 0.4 µl each primer (10 µM), 2.5 ng of sample cDNA, and 6.8 µl sterilized ultrapure H_2_O. The reaction programs were 30s at 95°C, 40 cycles of 95°C for 5s and 60°C for 34s. The results were analyzed using the ABI 7500 analysis software SDS 1.4. The qRT-PCR primers (Table S4) were designed using Beacon Designer 7.7 (PREMIER Biosoft International, CA, USA). The mRNA levels were measured by qRT-PCR using the SYBR Premix ExTaq™ (TaKaRa, Dalian, Liaoning, China). This was followed by the measurement of fluorescence during a 55 to 95°C melting curve in order to detect a single gene-specific peak and to check the absence of primer dimer peaks, and a single and discrete peak was detected for all primers tested. Negative controls were non-template reactions (replacing cDNA with H_2_O).

Expression levels of 16 genes were calculated relative to the reference gene *SinfGAPDH* using the Q-Gene method in Microsoft Excel-based software of Visual Basic [68], [69] For each sample, three biological replications were performed with each biological replication measured in three technique replications.

## Supporting Information

Figure S1 **Expression of** ***S. inferens*** **chemosensory transcripts in whole larvae body and different adult tissues.** GAPDH gene was used as a positive control and NC (no cDNA template) as a negative control. La, larvae whole body; PG, female pheromone glands; A, antennae; H, heads; T, thoraxes; Ab, abdomens (female without PG); L, legs; W, wings;♀, female, ♂, male. A, Expression of all chemosensory genes by using the first cDNA sample; B, Expression of 32 randomly chosen genes for checking the repeatability of the RT-PCR method by using the second cDNA sample.
(TIF)

Table S1 **The Blastx match of top 50 most abundant unigenes.** Except for the putative chemosensory genes in *S. inferens*.
(DOC)

Table S2 **Data of band intensity of RT-PCR products**. It is showing the repeatability of two biological replicates of 32 genes randomly chosen from the 92 ones. #: The band intensity was not calculated because of the irregular images, and were estimated by comparison with the normal bands.
(DOC)

Table S3 **Accession numbers for amino acid sequences of OBPs, CSPs and ORs used in phylogenetic analyses.**
(DOC)

Table S4 **Primers used for RT-PCR, qRT-PCR and RACE.**
(DOC)
