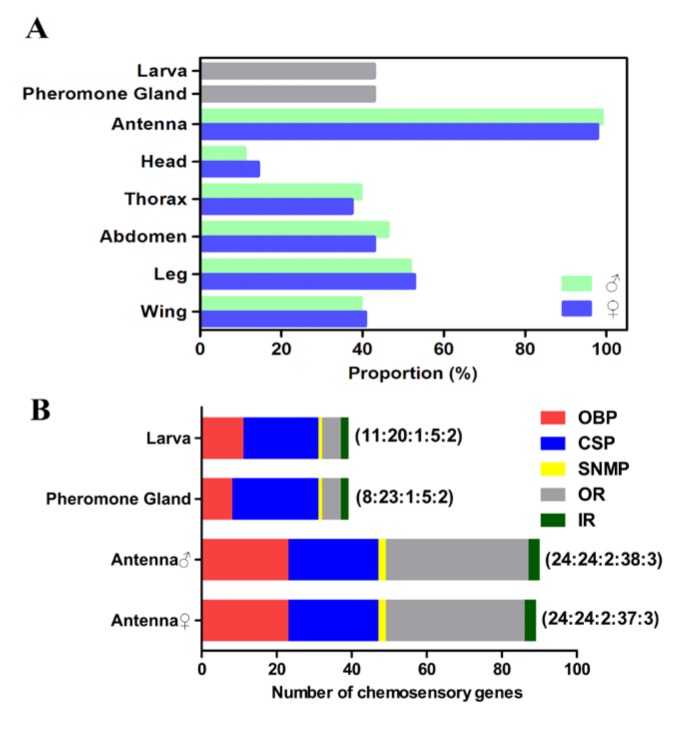# Correction: Differential Expression Patterns in Chemosensory and Non-Chemosensory Tissues of Putative Chemosensory Genes Identified by Transcriptome Analysis of Insect Pest the Purple Stem Borer *Sesamia inferens* (Walker)

**DOI:** 10.1371/annotation/0cd8f881-8121-4a54-88ff-e776f096e78a

**Published:** 2013-08-01

**Authors:** Ya-Nan Zhang, Jun-Yan Jin, Rong Jin, Yi-Han Xia, Jing-Jiang Zhou, Jian-Yu Deng, Shuang-Lin Dong

Figure 7 is currently a duplicate of Figure 6. Please see the correct version of Figure 7 at the following link:

**Figure:**