# Supplementary figures and images for: Differential Expression Patterns in Chemosensory and Non-Chemosensory Tissues of Putative Chemosensory Genes Identified by Transcriptome Analysis of Insect Pest the Purple Stem Borer Sesamia inferens (Walker)
Source: PLoS One. 2013 Jul 24;8(7):e69715. doi: 10.1371/journal.pone.0069715 (PMC3722147; doi:10.1371/journal.pone.0069715)

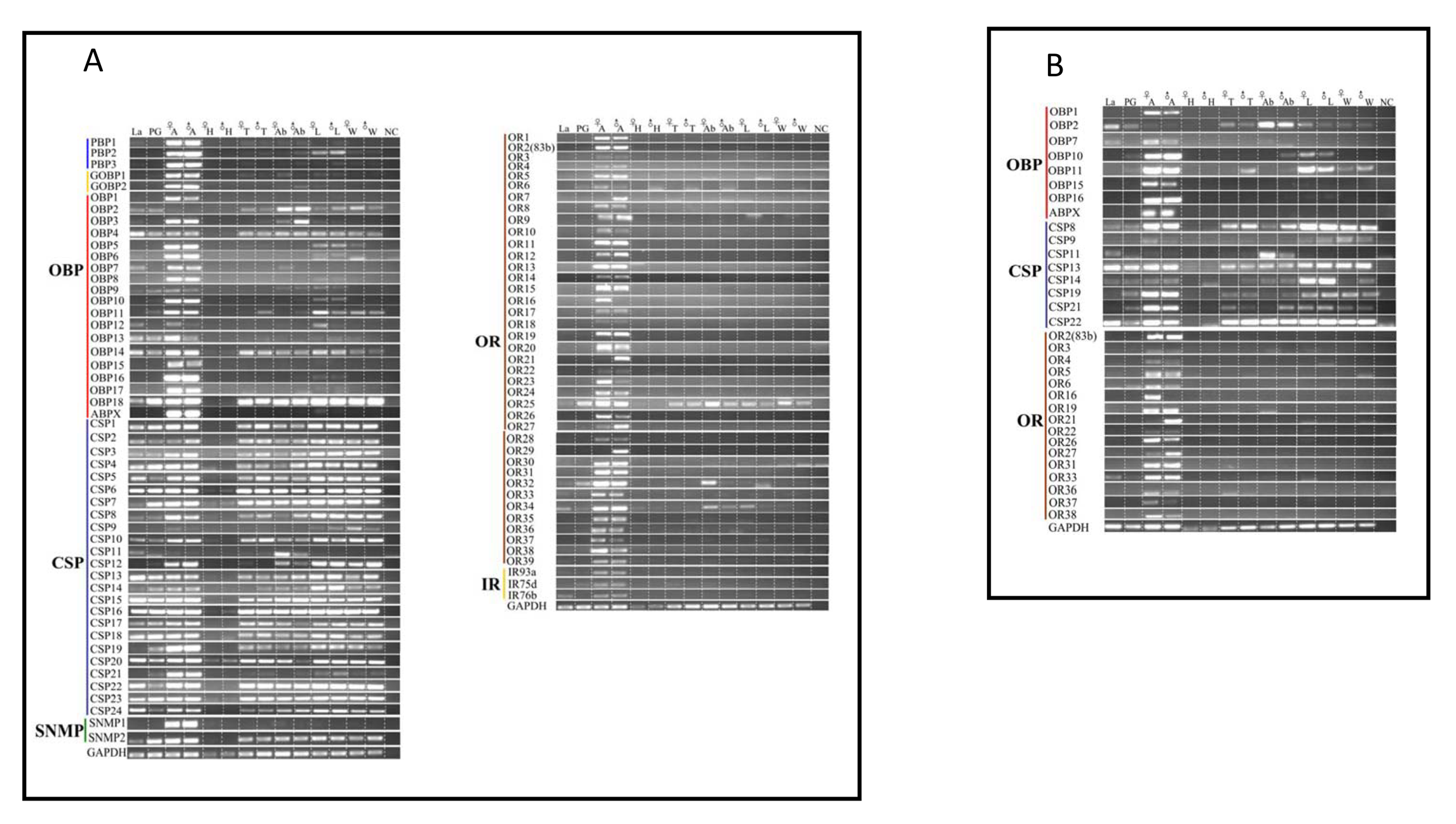

Supplement: Figure S1 — Expression of S. inferens chemosensory transcripts in whole larvae body and different adult tissues. GAPDH gene was used as a positive control and NC (no cDNA template) as a negative control. La, larvae whole body; PG, female pheromone glands; A, antennae; H, heads; T, thoraxes; Ab, abdomens (female without PG); L, legs; W, wings;♀, female, ♂, male. A, Expression of all chemosensory genes by using the first cDNA sample; B, Expression of 32 randomly chosen genes for checking the repeatability of the RT-PCR method by using the second cDNA sample. (TIF) [file pone.0069715.s001.tif]
