# Supplementary material for: Differential Expression Patterns in Chemosensory and Non-Chemosensory Tissues of Putative Chemosensory Genes Identified by Transcriptome Analysis of Insect Pest the Purple Stem Borer Sesamia inferens (Walker)
Source: PLoS One. 2013 Jul 24;8(7):e69715. doi: 10.1371/journal.pone.0069715 (PMC3722147; doi:10.1371/journal.pone.0069715)
Supplement: Table S1 — The Blastx match of top 50 most abundant unigenes. Except for the putative chemosensory genes in S. inferens. (DOC) [file pone.0069715.s002.doc]

**Table S1.** The Blastx match of top 50 most abundant unigenes except for the putative chemosensory genes in *S. inferens*.

| **Unigene**  **ID** | **ORF (bp)** | **Best Blastx Match** | | | | |
| --- | --- | --- | --- | --- | --- | --- |
|  |  | **Name** | **Acc. number** | **Species** | **E value** | **Identity (%)** |
| 2819 | 1131 | actin | XP_001659963.1 | [*Aedes aegypt*i] | 0.00 | 100 |
| 5085 | 618 | DUF233 protein | ACX53718.1 | [*Heliothis virescens*] | 1.00E-82 | 69 |
| 593 | -- | -- | -- | -- | -- | -- |
| 5086 | 612 | myosin light chain 2 | NP_001140188.1 | [*Antheraea pernyi*] | 8.00E-100 | 97 |
| 5094 | 369 | DUF233 protein | ACX53796.1 | [*Heliothis virescens*] | 4.00E-64 | 74 |
| 588 | 555 | muscle protein 20-like protein | BAG30780.1 | [*Papilio xuthus*] | 7.00E-118 | 89 |
| 606 | 369 | troponin C 2 | AEB54588.1 | [*Lonomia obliqua*] | 6.00E-79 | 97 |
| 605 | 107 | arginine kinase-like protein | ADC29631.1 | [*Plutella xylostella*] | 1.00E-73 | 97 |
| 5081 | 1161 | troponin T | BAG30738.1 | [*Papilio xuthus*] | 7.00E-166 | 95 |
| 2870 | -- | -- | -- | -- | -- | -- |
| 2830 | 599 | cytochrome c oxidase subunit III | YP_004734365.1 | [*Sesamia inferens*] | 2.00E-97 | 85 |
| 2839 | 552 | troponin I transcript variant C | ACN86370.1 | [*Bombyx mandarina*] | 2.00E-91 | 96 |
| 599 | 521 | cytochrome oxidase subunit I | ACF35272.1 | [*Copitarsia decolora*] | 3.00E-84 | 92 |
| 5088 | 735 | glutathione S-transferase | ACX47897.1 | [*Amyelois transitella*] | 3.00E-25 | 69 |
| 5079 | -- | -- | -- | -- | -- | -- |
| 5092 | 453 | myosin light chain | AEP43792.1 | [*Biston betularia*] | 2.00E-80 | 92 |
| 2867 | 562 | tropomyosin isoform 5 | ABF51445.1 | [*Bombyx mori*] | 6.00E-106 | 97 |
| 5120 | 308 | cytochrome c oxidase subunit 1 | BAG16746.1 | [*Neptunea arthritica*] | 3.00E-40 | 64 |
| 5125 | -- | -- | -- | -- | -- | -- |
| 608 | 80 | tropomyosin isoform 6 | ABF51400.1 | [*Bombyx mori*] | 100E-18 | 100 |
| 5114 | 614 | cytochrome oxidase subunit II | ADT91487.1 | [*Lepidoptera sp. VB-2010*] | 200E-82 | 88 |
| 2883 | 288 | muscle LIM protein | BAM18487.1 | [*Papilio xuthus*] | 8.00E-46 | 92 |
| 642 | -- | -- | -- | -- | -- | -- |
| 613 | -- | -- | -- | -- | -- | -- |
| 2853 | -- | -- | -- | -- | -- | -- |
| 610 | 345 | unknown secreted protein | BAM18659.1 | [*Papilio xuthus*] | 4.00E-61 | 63 |
| 2833 | 195 | Diapause-specific peptide | Q8T0W8.1 | [*Gastrophysa atrocyanea*] | 3.00E-21 | 72 |
| 607 | 297 | stress-sensitive B | BAM17721.1 | [*Papilio xuthus]* | 3.00E-74 | 97 |
| 5097 | 873 | heat shock cognate 70 | ACS36776.1 | [*Spodoptera exigua*] | 0.00 | 100 |
| 2869 | 237 | conserved hypothetical protein | BAM18983.1 | [*Papilio polytes*] | 2.00E-94 | 88 |
| 589 | 615 | muscle myosin heavy chain | BAG30740.1 | [*Papilio xuthus* ] | 8.00E-121 | 96 |
| 592 | -- | -- | -- | -- | -- | -- |
| 611 | -- | -- | -- | -- | -- | -- |

--, indicate that there is no results by Blastx in NCBI.
