# Supplementary material for: Differential Expression Patterns in Chemosensory and Non-Chemosensory Tissues of Putative Chemosensory Genes Identified by Transcriptome Analysis of Insect Pest the Purple Stem Borer Sesamia inferens (Walker)
Source: PLoS One. 2013 Jul 24;8(7):e69715. doi: 10.1371/journal.pone.0069715 (PMC3722147; doi:10.1371/journal.pone.0069715)
Supplement: Table S2 — Data of band intensity of RT-PCR products. It is showing the repeatability of two biological replicates of 32 genes randomly chosen from the 92 ones. #: The band intensity was not calculated because of the irregular images, and were estimated by comparison with the normal bands. (DOC) [file pone.0069715.s003.doc]

**Table S2.** Data of band intensity of RT-PCR products, showing the repeatability of two biological replicates of 32 genes randomly chosen from the 92 ones.

| **Gene** | **Replicate** | **Tissue** | | | | | | | | | | | | | | |
| --- | --- | --- | --- | --- | --- | --- | --- | --- | --- | --- | --- | --- | --- | --- | --- | --- |
|  |  | La | PG | A♀ | A♂ | | H♀ | H♂ | T♀ | T♂ | Ab♀ | Ab♂ | L♀ | L♂ | W♀ | W♂ |
| OBP1 | First |  |  | 1.16 | 0.77 | |  |  |  |  |  |  |  |  |  |  |
|  | Second# |  |  | 1.16 | 0.91 | |  |  |  |  |  |  |  |  |  |  |
|  | Mean |  |  | 1.16 | 0.84 | |  |  |  |  |  |  |  |  |  |  |
|  |  |  |  | *** | ** | |  |  |  |  |  |  |  |  |  |  |
|  | | | | | | | | | | | | | | | | |
| OBP2 | First | 0.66 | 0.72 | 0.55 | 0.52 | |  |  | 0.77 | 0.62 | 1.06 | 1.11 | 0.66 | 0.82 | 1.07 | 0.78 |
|  | Second | 1.02 | 0.60 | 0.44 | 0.42 | |  |  | 0.69 | 0.54 | 1.06 | 1.10 | 0.70 | 0.50 | 0.54 | 0.52 |
|  | Mean | 0.84 | 0.66 | 0.50 | 0.47 | |  |  | 0.73 | 0.58 | 1.06 | 1.11 | 0.68 | 0.66 | 0.81 | 0.65 |
|  |  | ** | ** | * | * | |  |  | ** | * | *** | *** | ** | ** | ** | ** |
|  | | | | | | | | | | | | | | | | |
| OBP7 | First | 1.31 |  | 1.62 | 1.38 | |  |  |  |  | 1.00 |  |  |  |  |  |
|  | Second | 0.70 |  | 0.85 | 0.72 | |  |  |  |  | 0.50 |  |  |  |  |  |
|  | Mean | 0.77 |  | 0.97 | 0.86 | |  |  |  |  | 0.55 |  |  |  |  |  |
|  |  | ** |  | ** | ** | |  |  |  |  | * |  |  |  |  |  |
|  | | | | | | | | | | | | | | | | |
| OBP10 | First |  | 0.39 | 1.16 | 1.16 | |  |  | 0.36 | 0.31 |  | 0.34 | 0.57 | 0.55 |  |  |
|  | Second |  | 0.45 | 1.16 | 1.16 | |  |  | 0.36 | 0.30 |  | 0.48 | 0.79 | 0.60 |  |  |
|  | Mean |  | 0.42 | 1.16 | 1.16 | |  |  | 0.36 | 0.31 |  | 0.41 | 0.68 | 0.58 |  |  |
|  |  |  | * | *** | *** | |  |  | * | * |  | * | ** | * |  |  |
|  | | | | | | | | | | | | | | | | |
| OBP11 | First |  | 0.50 | 1.16 | 1.15 | |  |  |  | 0.63 |  | 0.43 | 1.19 | 0.74 | 0.89 | 0.80 |
|  | Second |  | 0.51 | 1.16 | 1.16 | |  |  |  | 0.61 |  | 0.41 | 1.19 | 0.84 | 0.65 | 0.80 |
|  | Mean |  | 0.51 | 1.16 | 1.15 | |  |  |  | 0.62 |  | 0.42 | 1.19 | 0.79 | 0.77 | 0.80 |
|  |  |  | * | *** | *** | |  |  |  | ** |  | * | *** | ** | ** | ** |
|  | | | | | | | | | | | | | | | | |
| OBP15 | First |  |  | 1.04 | 0.84 | |  |  |  |  |  |  | 0.43 |  |  |  |
|  | Second |  |  | 1.02 | 0.82 | |  |  |  |  |  |  | 0.42 |  |  |  |
|  | Mean |  |  | 1.03 | 0.83 | |  |  |  |  |  |  | 0.42 |  |  |  |
|  |  |  |  | *** | ** | |  |  |  |  |  |  | * |  |  |  |
|  | | | | | | | | | | | | | | | | |
| OBP16 | First |  |  | 1.16 | 1.16 |  | |  |  |  |  |  | 0.33 | 0.35 |  |  |
|  | Second |  |  | 1.16 | 1.16 |  | |  |  |  |  |  | 0.35 | 0.37 |  |  |
|  | Mean |  |  | 1.16 | 1.16 |  | |  |  |  |  |  | 0.34 | 0.36 |  |  |
|  |  |  |  | *** | *** |  | |  |  |  |  |  | * | * |  |  |
|  | | | | | | | | | | | | | | | | |
| ABPX | First |  |  | 1.16 | 1.16 | |  |  |  |  |  |  | 0.49 | 0.35 |  |  |
|  | Second |  |  | 1.16 | 1.12 | |  |  |  |  |  |  | 0.42 | 0.41 |  |  |
|  | Mean |  |  | 1.16 | 1.14 | |  |  |  |  |  |  | 0.46 | 0.38 |  |  |
|  |  |  |  | *** | *** | |  |  |  |  |  |  | * | * |  |  |
|  | | | | | | | | | | | | | | | | |
| CSP8 | First | 0.44 | 0.45 | 1.16 | 1.00 | |  |  | 0.71 | 0.96 | 0.41 | 0.96 | 1.19 | 1.19 | 1.28 | 1.34 |
|  | Second | 0.59 | 0.57 | 1.16 | 1.16 | |  |  | 1.05 | 1.00 | 0.50 | 1.00 | 1.19 | 1.19 | 1.20 | 1.21 |
|  | Mean | 0.52 | 0.51 | 1.16 | 1.08 | |  |  | 0.88 | 0.98 | 0.45 | 0.98 | 1.19 | 1.19 | 1.24 | 1.28 |
|  |  | * | * | *** | *** | |  |  | ** | ** | * | ** | *** | *** | *** | *** |
|  |  |  |  |  |  | |  |  |  |  |  |  |  |  |  |  |
| CSP9 | First |  |  | 0.59 | 0.54 | |  |  |  |  |  |  | 0.57 | 0.67 | 0.93 | 0.70 |
|  | Second |  |  | 0.70 | 0.57 | |  |  |  |  |  |  | 0.62 | 0.72 | 0.87 | 0.72 |
|  | Mean |  |  | 0.64 | 0.56 | |  |  |  |  |  |  | 0.60 | 0.69 | 0.90 | 0.71 |
|  |  |  |  | ** | * | |  |  |  |  |  |  | ** | ** | ** | ** |
|  | | | | | | | | | | | | | | | | |
| CSP19 | First |  | 0.64 | 1.16 | 1.16 | |  |  | 0.76 | 0.58 | 0.37 | 0.43 | 0.93 | 0.93 | 0.91 | 0.56 |
|  | Second |  | 0.52 | 1.15 | 0.99 | |  |  | 0.48 | 0.43 | 0.33 | 0.40 | 0.61 | 0.73 | 0.72 | 0.64 |
|  | Mean |  | 0.58 | 1.15 | 1.08 | |  |  | 0.62 | 0.51 | 0.35 | 0.41 | 0.77 | 0.83 | 0.81 | 0.60 |
|  |  |  | * | *** | *** | |  |  | ** | * | * | * | ** | ** | ** | ** |
|  | | | | | | | | | | | | | | | | |
| CSP11 | First | 0.56 | 0.52 | 0.60 | 0.56 | |  |  |  | 0.56 | 1.06 | 0.77 |  |  |  |  |
|  | Second | 0.65 | 0.45 | 0.59 | 0.57 | |  |  |  | 0.52 | 1.06 | 0.78 |  |  |  |  |
|  | Mean | 0.61 | 0.49 | 0.60 | 0.57 | |  |  |  | 0.54 | 1.06 | 0.78 |  |  |  |  |
|  |  | ** | * | ** | * | |  |  |  | * | *** | ** |  |  |  |  |
|  | | | | | | | | | | | | | | | | |
| CSP13 | First | 1.19 | 0.91 | 1.16 | 1.16 | |  |  | 0.90 | 0.83 | 0.79 | 0.83 | 1.02 | 1.19 | 1.28 | 1.34 |
|  | Second | 1.19 | 1.09 | 1.16 | 1.16 | |  |  | 0.76 | 0.70 | 0.67 | 0.70 | 1.14 | 1.14 | 1.23 | 1.29 |
|  | Mean | 1.19 | 1.00 | 1.16 | 1.16 | |  |  | 0.83 | 0.76 | 0.73 | 0.76 | 1.08 | 1.16 | 1.25 | 1.32 |
|  |  | *** | *** | *** | *** | |  |  | ** | ** | ** | ** | *** | *** | *** | *** |
|  | | | | | | | | | | | | | | | | |
| CSP14 | First | 0.63 | 0.83 | 0.88 | 0.88 | |  |  |  | 0.68 | 0.69 | 0.70 | 1.19 | 1.19 | 0.97 | 0.95 |
|  | Second | 0.70 | 0.77 | 0.77 | 0.77 | |  |  |  | 0.70 | 0.71 | 0.74 | 1.19 | 1.19 | 0.55 | 0.89 |
|  | Mean | 0.66 | 0.80 | 0.83 | 0.83 | |  |  |  | 0.69 | 0.70 | 0.72 | 1.19 | 1.19 | 0.76 | 0.92 |
|  |  | ** | ** | ** | ** | |  |  |  | ** | ** | ** | *** | *** | ** | ** |
|  | | | | | | | | | | | | | | | | |
| CSP21 | First |  | 0.55 | 1.16 | 1.16 | |  |  | 0.48 | 0.46 |  | 0.45 | 0.56 | 0.69 | 0.50 | 0.54 |
|  | Second |  | 0.57 | 1.16 | 1.11 | |  |  | 0.66 | 0.47 |  | 0.70 | 0.77 | 0.81 | 0.63 | 0.60 |
|  | Mean |  | 0.56 | 1.16 | 1.14 | |  |  | 0.57 | 0.47 |  | 0.58 | 0.66 | 0.75 | 0.56 | 0.57 |
|  |  |  | * | *** | *** | |  |  | * | * |  | * | ** | ** | * | * |
|  | | | | | | | | | | | | | | | | |
| CSP22 | First | 1.19 | 0.72 | 1.16 | 1.16 | |  |  | 1.21 | 1.11 | 1.06 | 1.11 | 1.19 | 1.19 | 1.28 | 1.34 |
|  | Second | 1.19 | 0.64 | 1.16 | 1.16 | |  |  | 1.21 | 1.11 | 1.06 | 1.11 | 1.19 | 1.19 | 1.28 | 1.34 |
|  | Mean | 1.19 | 0.68 | 1.16 | 1.16 | |  |  | 1.21 | 1.11 | 1.06 | 1.11 | 1.19 | 1.19 | 1.28 | 1.34 |
|  |  | *** | ** | *** | *** | |  |  | *** | *** | *** | *** | *** | *** | *** | *** |
|  | | | | | | | | | | | | | | | | |
| OR2(83b) | First |  |  | 1.16 | 1.16 | |  |  |  |  |  |  |  |  |  |  |
|  | Second |  |  | 1.16 | 1.16 | |  |  |  |  |  |  |  |  |  |  |
|  | Mean |  |  | 1.16 | 1.16 | |  |  |  |  |  |  |  |  |  |  |
|  |  |  |  | *** | *** | |  |  |  |  |  |  |  |  |  |  |
|  | | | | | | | | | | | | | | | | |
| OR3 | First |  |  | 0.43 | 0.40 | |  |  |  |  |  |  |  |  |  |  |
|  | Second |  |  | 0.50 | 0.50 | |  |  |  |  |  |  |  |  |  |  |
|  | Mean |  |  | 0.46 | 0.45 | |  |  |  |  |  |  |  |  |  |  |
|  |  |  |  | * | * | |  |  |  |  |  |  |  |  |  |  |
|  | | | | | | | | | | | | | | | | |
| OR4 | First |  |  | 0.72 | 0.97 | |  |  |  |  |  |  |  |  |  |  |
|  | Second |  |  | 0.50 | 0.41 | |  |  |  |  |  |  |  |  |  |  |
|  | Mean |  |  | 0.61 | 0.69 | |  |  |  |  |  |  |  |  |  |  |
|  |  |  |  | ** | ** | |  |  |  |  |  |  |  |  |  |  |
|  | | | | | | | | | | | | | | | | |
| OR5 | First |  |  | 1.07 | 1.07 | |  |  |  |  |  |  |  |  |  |  |
|  | Second |  |  | 1.00 | 0.91 | |  |  |  |  |  |  |  |  |  |  |
|  | Mean |  |  | 1.03 | 0.99 | |  |  |  |  |  |  |  |  |  |  |
|  |  |  |  | *** | ** | |  |  |  |  |  |  |  |  |  |  |
|  | | | | | | | | | | | | | | | | |
| OR6 | First |  | 0.61 | 0.79 | 0.66 | |  |  |  |  |  |  |  |  |  |  |
|  | Second |  | 0.68 | 0.95 | 0.73 | |  |  |  |  |  |  |  |  |  |  |
|  | Mean |  | 0.65 | 0.87 | 0.69 | |  |  |  |  |  |  |  |  |  |  |
|  |  |  | ** | ** | ** | |  |  |  |  |  |  |  |  |  |  |
|  | | | | | | | | | | | | | | | | |
| OR16 | First |  |  | 1.16 |  | |  |  |  |  |  |  |  |  |  |  |
|  | Second |  |  | 1.16 |  | |  |  |  |  |  |  |  |  |  |  |
|  | Mean |  |  | 1.16 |  | |  |  |  |  |  |  |  |  |  |  |
|  |  |  |  | *** |  | |  |  |  |  |  |  |  |  |  |  |
|  | | | | | | | | | | | | | | | | |
| OR19 | First |  |  | 1.13 | 1.16 | |  |  |  |  |  |  |  |  |  |  |
|  | Second |  |  | 1.16 | 1.16 | |  |  |  |  |  |  |  |  |  |  |
|  | Mean |  |  | 1.15 | 1.16 | |  |  |  |  |  |  |  |  |  |  |
|  |  |  |  | *** | *** | |  |  |  |  |  |  |  |  |  |  |
|  | | | | | | | | | | | | | | | | |
| OR21 | First |  |  | 0.42 | 1.16 | |  |  |  |  |  |  |  |  |  |  |
|  | Second |  |  | 0.43 | 1.16 | |  |  |  |  |  |  |  |  |  |  |
|  | Mean |  |  | 0.42 | 1.16 | |  |  |  |  |  |  |  |  |  |  |
|  |  |  |  | * | *** | |  |  |  |  |  |  |  |  |  |  |
|  | | | | | | | | | | | | | | | | |
| OR22 | First |  |  | 0.52 | 0.51 | |  |  |  |  |  |  |  |  |  |  |
|  | Second |  |  | 0.55 | 0.58 | |  |  |  |  |  |  |  |  |  |  |
|  | Mean |  |  | 0.53 | 0.54 | |  |  |  |  |  |  |  |  |  |  |
|  |  |  |  | * | * | |  |  |  |  |  |  |  |  |  |  |
|  | | | | | | | | | | | | | | | | |
| OR26 | First |  |  | 1.16 | 0.73 | |  |  |  |  |  |  |  |  |  |  |
|  | Second |  |  | 1.16 | 0.68 | |  |  |  |  |  |  |  |  |  |  |
|  | Mean |  |  | 1.16 | 0.70 | |  |  |  |  |  |  |  |  |  |  |
|  |  |  |  | *** | ** | |  |  |  |  |  |  |  |  |  |  |
|  | | | | | | | | | | | | | | | | |
| OR27 | First |  |  | 0.66 | 1.11 | |  |  |  |  |  |  |  |  |  |  |
|  | Second |  |  | 0.64 | 1.09 | |  |  |  |  |  |  |  |  |  |  |
|  | Mean |  |  | 0.65 | 1.10 | |  |  |  |  |  |  |  |  |  |  |
|  |  |  |  | ** | *** | |  |  |  |  |  |  |  |  |  |  |
|  | | | | | | | | | | | | | | | | |
| OR31 | First |  |  | 1.16 | 1.16 | |  |  |  |  |  |  |  |  |  |  |
|  | Second |  |  | 1.16 | 1.16 | |  |  |  |  |  |  |  |  |  |  |
|  | Mean |  |  | 1.16 | 1.16 | |  |  |  |  |  |  |  |  |  |  |
|  |  |  |  | *** | *** | |  |  |  |  |  |  |  |  |  |  |
|  | | | | | | | | | | | | | | | | |
| OR33 | First | 0.59 | 0.55 | 1.08 | 0.93 | |  |  |  | 0.50 |  | 0.48 |  | 0.51 |  | 0.53 |
|  | Second | 0.75 | 0.55 | 1.16 | 1.16 | |  |  |  | 0.43 |  | 0.43 |  | 0.47 |  | 0.53 |
|  | Mean | 0.67 | 0.55 | 1.12 | 1.04 | |  |  |  | 0.47 |  | 0.46 |  | 0.49 |  | 0.53 |
|  |  | ** | * | *** | *** | |  |  |  | * |  | * |  | * |  | * |
|  | | | | | | | | | | | | | | | | |
| OR36 | First |  |  | 0.89 | 0.75 | |  |  |  |  |  |  |  |  |  |  |
|  | Second |  |  | 0.82 | 0.68 | |  |  |  |  |  |  |  |  |  |  |
|  | Mean |  |  | 0.85 | 0.72 | |  |  |  |  |  |  |  |  |  |  |
|  |  |  |  | ** | ** | |  |  |  |  |  |  |  |  |  |  |
|  | | | | | | | | | | | | | | | | |
| OR37 | First |  |  | 0.70 | 0.52 | |  |  |  |  |  |  |  |  |  |  |
|  | Second |  |  | 0.63 | 0.45 | |  |  |  |  |  |  |  |  |  |  |
|  | Mean |  |  | 0.66 | 0.49 | |  |  |  |  |  |  |  |  |  |  |
|  |  |  |  | ** | * | |  |  |  |  |  |  |  |  |  |  |
|  | | | | | | | | | | | | | | | | |
| OR38 | First |  |  | 1.16 | 0.77 | |  |  |  |  |  |  |  |  |  |  |
|  | Second |  |  | 1.16 | 0.84 | |  |  |  |  |  |  |  |  |  |  |
|  | Mean |  |  | 1.16 | 0.81 | |  |  |  |  |  |  |  |  |  |  |
|  |  |  |  | *** | ** | |  |  |  |  |  |  |  |  |  |  |
