# Supplementary material for: Differential Expression Patterns in Chemosensory and Non-Chemosensory Tissues of Putative Chemosensory Genes Identified by Transcriptome Analysis of Insect Pest the Purple Stem Borer Sesamia inferens (Walker)
Source: PLoS One. 2013 Jul 24;8(7):e69715. doi: 10.1371/journal.pone.0069715 (PMC3722147; doi:10.1371/journal.pone.0069715)
Supplement: Table S3 — Accession numbers for amino acid sequences of OBPs, CSPs and ORs used in phylogenetic analyses. (DOC) [file pone.0069715.s004.doc]

**Table S3** Accession numbers for amino acid sequences of OBPs, CSPs and ORs used in phylogenetic analyses.

| **Species** | **Protein name** | **Acc. number** | **Species** | **Protein name** | **Acc. number** |
| --- | --- | --- | --- | --- | --- |
| **OBPs** |  |  |  |  |  |
| ***Sesamia inferens*** | SinfPBP1 | JF927621.1 | ***Spodoptera littoralis*** | SlitEZ982114 | Legeai et al. 2011 |
|  | SinfPBP2 | JN984058.1 |  | SlitEZ982027 | Legeai et al. 2011 |
|  | SinfPBP3 | JF927622.1 |  | SlitFQ020630 | Legeai et al. 2011 |
|  | SinfGOBP1 | KC887506 |  | SlitFQ021928 | Legeai et al. 2011 |
|  | SinfGOBP2 | This study |  | SlitFQ014244 | Legeai et al. 2011 |
|  | SinfOBP1 | KC887507 |  | SlitFQ018693 | Legeai et al. 2011 |
|  | SinfOBP2 | KC887508 |  | SlitEZ982153 | Legeai et al. 2011 |
|  | SinfOBP3 | KC887509 | ***Bombyx mori*** | BmorOBP1 | Gong et al. 2009 |
|  | SinfOBP4 | KC887510 |  | BmorOBP2 | Gong et al. 2009 |
|  | SinfOBP5 | KC887511 |  | BmorOBP3 | Gong et al. 2009 |
|  | SinfOBP6 | KC887512 |  | BmorOBP4 | Gong et al. 2009 |
|  | SinfOBP7 | KC887513 |  | BmorOBP5 | Gong et al. 2009 |
|  | SinfOBP8 | KC887514 |  | BmorOBP6 | Gong et al. 2009 |
|  | SinfOBP9 | KC887515 |  | BmorOBP7 | Gong et al. 2009 |
|  | SinfOBP10 | KC887516 |  | BmorOBP8 | Gong et al. 2009 |
|  | SinfOBP12 | KC887518 |  | BmorOBP9 | Gong et al. 2009 |
|  | SinfOBP13 | KC887519 |  | BmorOBP10 | Gong et al. 2009 |
|  | SinfOBP14 |  |  | BmorOBP11 | Gong et al. 2009 |
|  | SinfOBP15 | KC887521 |  | BmorOBP12 | Gong et al. 2009 |
|  | SinfOBP16 | KC887522 |  | BmorOBP13 | Gong et al. 2009 |
|  | SinfOBP17 | KC887523 |  | BmorOBP14 | Gong et al. 2009 |
|  | SinfOBP18 |  |  | BmorOBP15 | Gong et al. 2009 |
|  | SinfABPX | KC887520 |  | BmorOBP16 | Gong et al. 2009 |
| ***Manduca sexta*** | MsexABP2 | Grosse-Wilde et al. 2011 |  | BmorOBP17 | Gong et al. 2009 |
|  | MsexABP3 | Grosse-Wilde et al. 2011 |  | BmorOBP18 | Gong et al. 2009 |
|  | MsexABP4 | Grosse-Wilde et al. 2011 |  | BmorOBP19 | Gong et al. 2009 |
|  | MsexABP5 | Grosse-Wilde et al. 2011 |  | BmorOBP20 | Gong et al. 2009 |
|  | MsexABP6 | Grosse-Wilde et al. 2011 |  | BmorOBP21 | Gong et al. 2009 |
|  | MsexABP7 | Grosse-Wilde et al. 2011 |  | BmorOBP22 | Gong et al. 2009 |
|  | MsexABP8 | Grosse-Wilde et al. 2011 |  | BmorOBP23 | Gong et al. 2009 |
|  | MsexOBP | Grosse-Wilde et al. 2011 |  | BmorOBP25 | Gong et al. 2009 |
|  | MsexOBP2 | Grosse-Wilde et al. 2011 |  | BmorOBP26 | Gong et al. 2009 |
|  | MsexOBP3 | Grosse-Wilde et al. 2011 |  | BmorOBP27 | Gong et al. 2009 |
|  | MsexOBP4 | Grosse-Wilde et al. 2011 |  | BmorOBP28 | Gong et al. 2009 |
|  | MsexOBP5 | Grosse-Wilde et al. 2011 |  | BmorOBP29 | Gong et al. 2009 |
|  | MsexOBP6 | Grosse-Wilde et al. 2011 |  | BmorOBP30 | Gong et al. 2009 |
|  | MsexOBP7 | Grosse-Wilde et al. 2011 |  | BmorOBP31 | Gong et al. 2009 |
|  | MsexGOBP1 | Grosse-Wilde et al. 2011 |  | BmorOBP32 | Gong et al. 2009 |
|  | MsexGOBP2 | Grosse-Wilde et al. 2011 |  | BmorOBP33 | Gong et al. 2009 |
|  | MsexPBP1 | Grosse-Wilde et al. 2011 |  | BmorOBP34 | Gong et al. 2009 |
|  | MsexPBP2 | Grosse-Wilde et al. 2011 |  | BmorOBP35 | Gong et al. 2009 |
|  | MsexPBP3 | Grosse-Wilde et al. 2011 |  | BmorOBP36 | Gong et al. 2009 |
| ***Spodoptera littoralis*** | SlitFQ017058 | Legeai et al. 2011 |  | BmorOBP37 | Gong et al. 2009 |
|  | SlitFQ031480 | Legeai et al. 2011 |  | BmorOBP38 | Gong et al. 2009 |
|  | SlitFQ019778 | Legeai et al. 2011 |  | BmorOBP39 | Gong et al. 2009 |
|  | SlitFQ021259 | Legeai et al. 2011 |  | BmorOBP40 | Gong et al. 2009 |
|  | SlitFQ021918 | Legeai et al. 2011 |  | BmorOBP41 | Gong et al. 2009 |
|  | SlitEZ983259 | Legeai et al. 2011 |  | BmorOBP42 | Gong et al. 2009 |
|  | SlitGW825701 | Legeai et al. 2011 |  | BmorOBP43 | Gong et al. 2009 |
|  | SlitFQ021292 | Legeai et al. 2011 |  | BmorOBP44 | Gong et al. 2009 |
| **CSPs** |  |  |  |  |  |
| ***Sesamia inferens*** | SinfCSP1 |  | ***Manduca sexta*** | MsexSAP2 | Grosse-Wilde et al. 2011 |
|  | SinfCSP3 |  |  | MsexSAP3 | Grosse-Wilde et al. 2011 |
|  | SinfCSP4 | KC907741 |  | MsexSAP4 | Grosse-Wilde et al. 2011 |
|  | SinfCSP5 |  |  | MsexSAP5 | Grosse-Wilde et al. 2011 |
|  | SinfCSP6 | KC907742 | ***Spodoptera littoralis*** | SlitFQ024845 | Legeai et al. 2011 |
|  | SinfCSP8 | KC907744 |  | SlitEZ981604 | Legeai et al. 2011 |
|  | SinfCSP10 | KC907746 |  | SlitEZ983373 | Legeai et al. 2011 |
|  | SinfCSP12 | KC907748 |  | SlitEZ982930 | Legeai et al. 2011 |
|  | SinfCSP13 | KC907749 |  | SlitEZ982103 | Legeai et al. 2011 |
|  | SinfCSP14 | KC907750 |  | SlitEZ982609 | Legeai et al. 2011 |
|  | SinfCSP15 | KC907751 |  | SlitEZ983355 | Legeai et al. 2011 |
|  | SinfCSP16 | KC907752 |  | SlitGW825956 | Legeai et al. 2011 |
|  | SinfCSP17 | KC907753 |  | SlitHO118383 | Legeai et al. 2011 |
|  | SinfCSP18 | KC907754 | ***Bombyx mori*** | BmorCSP1 | FJ425876.1 |
|  | SinfCSP19 | KC907755 |  | BmorCSP2 | NP_001091778 |
|  | SinfCSP20 | KC907756 |  | BmorCSP3 | NM_001043598.2 |
|  | SinfCSP21 | KC907757 |  | BmorCSP5 | NM_001043597.1 |
|  | SinfCSP22 | KC907758 |  | BmorCSP6 | NM_001043935.2 |
|  | SinfCSP23 | KC907759 |  | BmorCSP7 | NM_001043603.1 |
|  | SinfCSP24 | KC907760 |  | BmorCSP8 | NM_001043602.1 |
| ***Manduca sexta*** | MsexCSP1 | Grosse-Wilde et al. 2011 |  | BmorCSP9 | NP_001037069.1 |
|  | MsexCSP3 | Grosse-Wilde et al. 2011 |  | BmorCSP10 | NM_001043599.1 |
|  | MsexCSP4 | Grosse-Wilde et al. 2011 |  | BmorCSP11 | NM_001098309.1 |
|  | MsexCSP5 | Grosse-Wilde et al. 2011 |  | BmorCSP12 | NM_001098310.1 |
|  | MsexCSP10 | Grosse-Wilde et al. 2011 |  | BmorCSP13 | NM_001043715.1 |
|  | MsexCSP11 | Grosse-Wilde et al. 2011 |  | BmorCSP14 | NM_001043727.2 |
|  | MsexCSP13 | Grosse-Wilde et al. 2011 |  | BmorCSP15 | NM_001098311.1 |
|  | MsexCSP14 | Grosse-Wilde et al. 2011 |  | BmorCSP16 | NM_001098312.1 |
|  | MsexSAP1 | Grosse-Wilde et al. 2011 |  |  |  |
| **ORs** |  |  |  |  |  |
| ***Sesamia inferens*** | SinfOR1 | KC960453 | ***Heliothis virescenss*** | HvirOR11 | CAG38112 |
|  | SinfOR2 | KC960454 |  | HvirOR12 | CAG38113 |
|  | SinfOR9 | KC960459 |  | HvirOR13 | CAG38114 |
|  | SinfOR18 | KF008006 |  | HvirOR14 | CAG38115 |
|  | SinfOR19 | KC960466 |  | HvirOR15 | CAG38116 |
|  | SinfOR20 | KC960467 |  | HvirOR16 | CAG38117 |
|  | SinfOR21 | KC960468 |  | HvirOR17 | CAG38118 |
|  | SinfOR23 | KC960470 |  | HvirOR18 | CAG38119 |
|  | SinfOR25 | KC960472 |  | HvirOR19 | CAG38120 |
|  | SinfOR26 | KC960473 |  | HvirOR20 | CAG38121 |
|  | SinfOR27 | KC960474 |  | HvirOR21 | CAG38122 |
|  | SinfOR28 | KC960475 | ***Bombyx mori*** | BmorOR1 | BAD69584 |
|  | SinfOR29 | KC960476 |  | BmorOR2 | BAD69585 |
|  | SinfOR30 | KC960477 |  | BmorOR3 | BAD89567 |
|  | SinfOR31 | KC960478 |  | BmorOR4 | BAD89568 |
|  | SinfOR32 | KC960479 |  | BmorOR5 | BAD89569 |
|  | SinfOR33 | KC960480 |  | BmorOR6 | BAD89570 |
|  | SinfOR35 | KC960481 |  | BmorOR7 | NP_001106227 |
|  | SinfOR36 | KC960482 |  | BmorOR8 | BAH66308 |
|  | SinfOR38 | KC960483 |  | BmorOR9 | BAH66309 |
|  | SinfOR39 | KC960484 |  | BmorOR10 | DAA05970 |
| ***Manduca sexta*** | MsexOR1 | Grosse-Wilde et al. 2011 |  | BmorOR11 | BAH66310 |
|  | MsexOR2 | Grosse-Wilde et al. 2011 |  | BmorOR12 | BAH66311 |
|  | MsexOR4 | Grosse-Wilde et al. 2011 |  | BmorOR13 | BAH66312 |
|  | MsexOR5 | Grosse-Wilde et al. 2011 |  | BmorOR14 | BAH66313 |
|  | MsexOR6 | Grosse-Wilde et al. 2011 |  | BmorOR15 | DAA05974 |
|  | MsexOR7 | Grosse-Wilde et al. 2011 |  | BmorOR16 | BAH66314 |
|  | MsexOR8 | Grosse-Wilde et al. 2011 |  | BmorOR17 | BAH66315 |
|  | MsexOR9 | Grosse-Wilde et al. 2011 |  | BmorOR18 | BAH66316 |
|  | MsexOR10 | Grosse-Wilde et al. 2011 |  | BmorOR19 | DAA05977 |
|  | MsexOR11 | Grosse-Wilde et al. 2011 |  | BmorOR20 | BAH66317 |
|  | MsexOR12 | Grosse-Wilde et al. 2011 |  | BmorOR21 | BAH66318 |
|  | MsexOR14 | Grosse-Wilde et al. 2011 |  | BmorOR22 | BAH66319 |
|  | MsexOR16 | Grosse-Wilde et al. 2011 |  | BmorOR23 | BAH66320 |
|  | MsexOR17 | Grosse-Wilde et al. 2011 |  | BmorOR24 | BAH66321 |
|  | MsexOR18 | Grosse-Wilde et al. 2011 |  | BmorOR25 | BAH66322 |
|  | MsexOR20 | Grosse-Wilde et al. 2011 |  | BmorOR26 | BAH66323 |
|  | MsexOR21 | Grosse-Wilde et al. 2011 |  | BmorOR27 | BAH66324 |
|  | MsexOR22 | Grosse-Wilde et al. 2011 |  | BmorOR28 | BAH66325 |
|  | MsexOR23 | Grosse-Wilde et al. 2011 |  | BmorOR29 | BAH66326 |
|  | MsexOR24 | Grosse-Wilde et al. 2011 |  | BmorOR30 | BAH66327 |
|  | MsexOR25 | Grosse-Wilde et al. 2011 |  | BmorOR32 | BAH66328 |
|  | MsexOR26 | Grosse-Wilde et al. 2011 |  | BmorOR33 | BAH66329 |
|  | MsexOR27 | Grosse-Wilde et al. 2011 |  | BmorOR35 | BAH66332 |
|  | MsexOR28 | Grosse-Wilde et al. 2011 |  | BmorOR36 | BAH66333 |
|  | MsexOR29 | Grosse-Wilde et al. 2011 |  | BmorOR37 | BAH66334 |
|  | MsexOR30 | Grosse-Wilde et al. 2011 |  | BmorOR38 | BAH66335 |
|  | MsexOR31 | Grosse-Wilde et al. 2011 |  | BmorOR39 | BAH66336 |
|  | MsexOR32 | Grosse-Wilde et al. 2011 |  | BmorOR40 | BAH66337 |
|  | MsexOR33 | Grosse-Wilde et al. 2011 |  | BmorOR41 | DAA05997 |
|  | MsexOR34 | Grosse-Wilde et al. 2011 |  | BmorOR42 | BAH66338 |
|  | MsexOR35 | Grosse-Wilde et al. 2011 |  | BmorOR44 | BAH66339 |
|  | MsexOR36 | Grosse-Wilde et al. 2011 |  | BmorOR45 | BAH66340 |
|  | MsexOR37 | Grosse-Wilde et al. 2011 |  | BmorOR46 | BAH66341 |
|  | MsexOR38 | Grosse-Wilde et al. 2011 |  | BmorOR47 | BAH66342 |
|  | MsexOR39 | Grosse-Wilde et al. 2011 |  | BmorOR50 | BAH66345 |
|  | MsexOR40 | Grosse-Wilde et al. 2011 |  | BmorOR51 | BAH66346 |
|  | MsexOR41 | Grosse-Wilde et al. 2011 |  | BmorOR53 | BAH66347 |
|  | MsexOR42 | Grosse-Wilde et al. 2011 |  | BmorOR54 | BAH66348 |
|  | MsexOR43 | Grosse-Wilde et al. 2011 |  | BmorOR55 | BAH66349 |
|  | MsexOR44 | Grosse-Wilde et al. 2011 |  | BmorOR56 | BAH66350 |
|  | MsexOR45 | Grosse-Wilde et al. 2011 |  | BmorOR57 | BAH66351 |
|  | MsexOR46 | Grosse-Wilde et al. 2011 |  | BmorOR58 | BAH66352 |
|  | MsexOR47 | Grosse-Wilde et al. 2011 |  | BmorOR59 | BAH66353 |
| ***Heliothis virescenss*** | HvirOR1 | CAD31850 |  | BmorOR60 | BAH66354 |
|  | HvirOR2 | CAD31851 |  | BmorOR61 | BAH66355 |
|  | HvirOR3 | CAD31852 |  | BmorOR62 | BAH66357 |
|  | HvirOR4 | CAD31946 |  | BmorOR63 | BAH66358 |
|  | HvirOR5 | CAD31947 |  | BmorOR64 | BAH66359 |
|  | HvirOR6 | CAD31948 |  | BmorOR65 | BAH66360 |
|  | HvirOR7 | CAD31853 |  | BmorOR66 | BAH66361 |
|  | HvirOR8 | CAD31949 |  | BmorOR67 | BAH66362 |
|  | HvirOR9 | CAD31950 |  | BmorOR68 | BAH66363 |
|  | HvirOR10 | CAG38111 |  |  |  |

Note: PBP1, PBP2, PBP3 and GOBP2 were previously deposited by others. Genes with obtained fragments less than 200 bp in length were not able to be deposited in the GenBank, and thus no accession number is provided.
