# Supplementary material for: Differential Expression Patterns in Chemosensory and Non-Chemosensory Tissues of Putative Chemosensory Genes Identified by Transcriptome Analysis of Insect Pest the Purple Stem Borer Sesamia inferens (Walker)
Source: PLoS One. 2013 Jul 24;8(7):e69715. doi: 10.1371/journal.pone.0069715 (PMC3722147; doi:10.1371/journal.pone.0069715)
Supplement: Table S4 — Primers used for RT-PCR, qRT-PCR and RACE. (DOC) [file pone.0069715.s005.doc]

**Table S4** Primers used for RT-PCR, qRT-PCR and RACE.

| **Purpose** | **Forward (5'-3')** | **Reverse (5'-3')** | **Purpose** | **Forward (5'-3')** | **Reverse (5'-3')** |
| --- | --- | --- | --- | --- | --- |
| **RT-PCR** |  |  | **RT-PCR** |  |  |
| PBP1 | TCAGGGTTCTCGAAGGGTTTG | CTTGGACGCCATGCACATTAC | OR18 | TGGTTCTTCTGGCAATCA | GATATGAAGGTTCCATAGGTAAG |
| PBP2 | TGATCCACGGCTGTATG | CAAGACTTCTCCCACGAT | OR19 | GGTCTATCAATACAAGGTTACG | CACTGCTGCCAAATACTC |
| PBP3 | GACATCATCCACGACTGT | GTGAAGACCTCGCTCAT | OR20 | GGTTGAAGGATTGTCACAGGAG | TGATTTGATAAGCAGTGACGCA |
| GOBP1 | TTGACCGAGGAGAAGATG | GAGTCTGTGAGCAGGTTA | OR21 | ATTGGTCGGAAGTGAGAG | CATAGAAGTAACTCCTACATTAGC |
| GOBP2 | CTGTATGTCCAACAAGTTCTC | CTGTCGTATTGCTTCTCAC | OR17 | CCACTATACAACTAATGCTACAG | TTGAAGATGGCGTATGATTG |
| OBP1 | AGAAGTCTGCTTGTATCGTA | GCTTGTGATTTACCTCTCTG | OR22 | TATGTTGGGCACCAGTAA | CAGATTCGTCGTTGTCATC |
| OBP2 | CTACGCTGACCTCTTGAG | TGGCAGTTTAGGGTTAGAA | OR23 | CAGTTGCTGTCAGGTTGAAATG | TGTCGTGGAATCATCATACTGG |
| OBP3 | AACGACTGGTGAATGCTA | CTTGAAGCCGAACTTTGG | OR24 | CCGAAAGAACCAGAACATAG | CAGTTAGAGGCATTATTATTCCA |
| OBP4 | TCCAATGTCTCAGCTCTC | GTCAGTCTGTCACACTCA | OR25 | ACCCTCATCTGCCACGAACT | GCTCCCAACCACAGCCATA |
| OBP5 | GTTAGCAGTGTCGTGATG | TGGTCTTCTGTTACTTGGT | OR26 | ATGCCTTGTTACTACAGCGACC | TATTCCCAGGCGGCATTC |
| OBP6 | GATCATCCTCTATCATTGTCAG | ATTCCTCTGTTGTCCATCA | OR27 | TTACGGACCACTAACAGTAG | TTGCCACGGAATACAATATG |
| OBP7 | CGTGATGGATGACAAAGG | ACAGATTCGCCGTTAATATG | OR28 | TAAACGGGACACAAAGGA | GTTGGAACAGCAGATATGG |
| OBP8 | GCAGCCTCAGTGGCAATAAC | TCACAGCCTTTCTCGCCAT | OR29 | GCTCACAAATGACGACGGC | TCCCACGGTACACCATAGACTG |
| OBP9 | CCGACAACTATCCAATAACAG | GCCTTCAACCGATAATTCTC | OR30 | AAGTAGGTGAGGCGATATAC | AGTGAGGGAGAGGTTGTA |
| OBP10 | ATAGTATCTGAAGACGGACTC | GCATCTCAGGAACTTGTAAG | OR31 | CTTCGCTCCTCTGATCTG | CACCTGTCTCTGTTGTTTC |
| OBP11 | TGCGAAGACTGACGATTA | GTTATAGTACAGGTTGGTTCTC | OR32 | ACAACTCCGCATTATGAAC | GCAATGTGTAATAACTGTAAGC |
| OBP12 | GGTAGTGCCGACTCTATC | AACTCCTCATCGTTGTTTAC | OR33 | GTCTGATGATTCGGTCTGA | TTGAAGTAGGAGTAGGATGAG |
| OBP13 | GCGATACGATCTGAACAC | GCTTACTTCTTGGTCATTAACT | OR34 | TTCCATTGCCTGCTAATAAC | TAATAAGTCGGTGATAATCTATGC |
| OBP14 | GACTACAAGACTGACAATGAG | TTAGGAACCTTAGCGAGAG | OR35 | GCTGATCGTCATTACTATGC | GATAGACACGTAGGATTGGTA |
| OBP15 | ATGACTCGGTCTCAACTTAA | TGACATAGCACATTACATTCC | OR36 | TGGCGATATTACTGTAGAGG | TGAAGATTGTCCGTAGCA |
| OBP16 | CAGAGGAGGATATAACGAATTG | AGTGCCATCATCGTCTAC | OR37 | TGGTGGTCTTGCTGAAACGAT | GCTGCGCCCGGATTATTATAG |
| OBP17 | GATGAAGGCTCCTGTGAA | TTGCTGTCCAATAAGATGC | OR38 | GTGCTATCTCTCGGGATC | CATCAGGTTGTTGTGGAAG |
| OBP18 | TCCGTCAGTCACAGGTTTGTCAT | GGGAATACGATGTAGATACCGCTC | OR39 | CTGCTGGTGGTATCATAATG | GTGTCATAATCTACTCGCTTAC |
| ABPX | GTGGAGATGGATGAGGAC | CACTTGATGTAGCACTTGAG | IR93a | CAGTCCGTATCTTCCAGTAA | CGTGTGATTGTTGACCTC |
| CSP1 | GTACGAGTACCCAGATGAG | ATAGAGTTAAAGTGTTCCTTAGC | IR75d | GCCTATCATAGCGTGGTA | CTGACTTATTATGTCGTACCC |
| CSP2 | CTATCCGTGGTCGTGATG | GGATCTCGTCAAGGTCTAC | IR76b | ACCTTACTGCCTTCCTTAC | TCGCCTTCAGATTTAACAAC |
| CSP3 | CAACAACGAGTCAGAACAC | TTAAGCCTTGATCTCTTTGAG | GAPDH | CCATTAACGACCCTTTCATC | ACGAGGTGTCCATCTTG |
| CSP4 | CTGCCTTTAAGAAACAAATCC | TTGGAAGCCCTTGACTAC | **qRT-PCR** |  |  |
| CSP5 | AGGAGCACATCAGAGAAG | TTCCAGTAATCTGCCTCG | OBP3 | AACGACTGGTGAATGCTA | CTTGAAGCCGAACTTTGG |
| CSP6 | AGGAGCACATCAGAGAAG | GTCGTACTTGGCAGTCA | ABPX | GATGGATGAGGACATGGC | GCACTTGATGTAGCACTTG |
| CSP7 | TGGCTCAAGAAGTGAAGAA | CACGCCTGTTGGTATTTG | GOBP1 | TTGACCGAGGAGAAGATG | GAGTCTGTGAGCAGGTTA |
| CSP8 | AAGGAACATTTCAAGGAAGC | CATTCTCGTGGTTGATTAGG | GOBP2 | CTGTATGTCCAACAAGTTCTC | CTGTCGTATTGCTTCTCAC |
| CSP9 | TGCGTGATGGATAAGGGACC | CGAGGTTCGCTCTTTGATTTG | CSP8 | AAGGAACATTTCAAGGAAGC | CATTCTCGTGGTTGATTAGG |
| CSP10 | CCGAAATATGACCAGAGATATG | ATCCACGTAGTTCCTGAC | CSP9 | TACGAGGGATGGAAAGAAC | TTGATTTGATGCCAAGGAG |
| CSP11 | CAGTGTTCGATGCAAGAA | AAATTAAATTCAGCCGTATTGG | CSP11 | CAGTGTTCGATGCAAGAA | AAATTAAATTCAGCCGTATTGG |
| CSP12 | CACTCTTTTAAAACTACCATTGGCA | TGGATAGCCCACTAATCCAGGA | CSP24 | CTGCTTCTTGGACAAAGG | GATGTTAGTCTTCTGCTTCTC |
| CSP13 | AAGCAAGAGCAGTCTATCA | TTGTCGCACAGTTGTTTC | SNMP1 | AGACCAACCGCTACATAG | GCATAGAGGCGTACATTG |
| CSP14 | GACGCTCAGAAACACATC | TCGCTTCCTGGATCATAC | SNMP2 | TTCAGACTCCTCCATATTCC | GCTGATGTTGAACTTCTCC |
| CSP15 | CGATATTACTATGTTGCGTGGC | GCTGAGGGAAGTTTCGTTGG | IR93a | CAGTCCGTATCTTCCAGTAA | CGTGTGATTGTTGACCTC |
| CSP16 | CCATCGCTCGTCCTGAAGA | GTCGGACAGTTGCTTCCAGTAG | IR76b | ACCTTACTGCCTTCCTTAC | TCGCCTTCAGATTTAACAAC |
| CSP17 | TCTCACCTCAAAGAAGCAT | TAGTCAGCCTCGTTGTTG | OR2(OR83b) | TCGGCTACCTGTGCTATA | CGTACCATTGGCAAGAGTA |
| CSP18 | TAGAGGAGGACTGTGCTA | TTCGTGATTGATAAGATGGC | OR21 | ATTGGTCGGAAGTGAGAG | CATAGAAGTAACTCCTACATTAGC |
| CSP19 | GGCAGTGATAGACAGACA | CTTCGGCTAGAAACTTATCG | OR23 | CAGTTGCTGTCAGGTTGAAATG | TGTCGTGGAATCATCATACTGG |
| CSP20 | ACACCGACAAGTACGATAA | CTCAATGGCATCCGAAAG | OR29 | ATTAGGACTTGCTGATGTTG | TGCTATTGAGGAAGGTGAA |
| CSP21 | GATGCTGCTAATATACTTGACA | GGCGTTCATTGTTCAGAA | GAPDH | CCATTAACGACCCTTTCATC | ACGAGGTGTCCATCTTG |
| CSP22 | ATACGACCACATCAACATTG | TCCTTCAGGAGTACATCTTC | **RACE** | **5'(5'-3')** | **3'(5'-3')** |
| CSP23 | GCCCTAAAGGAGACTCTG | TCTGGACGCTTATTCACTAA | CSP6-GSP | GGTTCCCTTCTTCTGGGCTTCATTGC | AACCGCCGCCTACTGGTGCCCTA |
| CSP24 | CTGCTTCTTGGACAAAGG | GATGTTAGTCTTCTGCTTCTC | CSP6-NGSP | CATTGTCGTAACGGTCGGTGTACTGAG | CGCCAAATGCAATGAAGCCCAGAA |
| SNMP1 | AGACCAACCGCTACATAG | GCATAGAGGCGTACATTG | CSP12-GSP | GAACCCGCGAGACGCAGCTAGTTAGTA | GCGGTGACTTCAAGCAGGTGGCAG |
| SNMP2 | TTCAGACTCCTCCATATTCC | GCTGATGTTGAACTTCTCC | CSP12-NGSP | GAAGTCACCGCAATCCCCTTTTTCC | ATCGAAGCCAAACAGGAATAACGCTC |
| OR1 | CTACTCTGATTTCCTCCTCTGCA | GACTTATCCCTCCGAACAATCA | CSP14-GSP | GTTTTGACCGCATCCGGGATGTCCT | AAAGGACATCCCGGATGCGGTCAA |
| OR2(OR83b) | AGCAGCCTACTCTTGCCAATG | CTTGAGTTGCACCAGCACCAT | CSP14-NGSP | ACGGCGTCACAGGTCACTCTGTCCA | GCTGCACAAAATGTACGGACGCTCA |
| OR3 | GAGCAAGACACCAAGTTC | CGAGACAAGGAGATGTAGT | CSP15-GSP | CACTGCTGAGGGAAGTTTCGTTGGA | ACCCTGCGACCCCATCGGAAAAC |
| OR4 | ACTCTCACTGGACACATAC | TCTTCCTTTGTTCCTGTTTC | CSP15-NGSP | CAAGAGTCTTTAGCCGTTTTCCGATG | CGTCCAACGAAACTTCCCTCAGCA |
| OR5 | TGCCGTTGGTATCTAGC | AATCCAGAAGCCGTCAG | CSP16-GSP | TTGGCGTATTTACCTTCCGGGTCAT | TCTTACATCAAGTGCGGCCTGGACC |
| OR6 | AGAGGAAAGTTCGGAGATAG | CGCACCATGATTATGAGC | CSP16-NGSP | CTCCTTCTGTTTGTCGGTGCACTTCG | GCATTGGAAACTCATTGCGCGAAGT |
| OR7 | ATGTTTAACTTGATTCCAATGC | ATTCGTCTCATAATTCCAAGG | CSP18-GSP | AGTCCTCCTCTAACGCTTCCCTGATGT | CTGTGTCTATTCGGGCTTGTGATGG |
| OR8 | AGAGAGATAGTGTCCGAATG | CGATAATGACGATCCAGAAC | CSP18-NGSP | TCAGCAGGCGACGATTACCAAGGA | TCCTTGGTAATCGTCGCCTGCTGA |
| OR9 | AACTCCTGCTCTTCTACTG | CCTTCGTATCTTCTTGTTAGC | CSP20-GSP | TCCTCCAGGTGCCAGTGGGATCA | CTGGATGAGATCCTCTCCAACAAACG |
| OR10 | TGCATACCCAGATGTCAC | AAGGAGACGACCAGAATG | CSP20-NGSP | CCTTCTCTTGCGCCTCCGTGCAT | CTGCGTCTTGGACAAAGGAAAGTGCA |
| OR11 | GATGTCGCAGATTCAAGAG | AAGAGTTCATTATCGCAGTG | CSP21-GSP | GCAGGCGTTCATTGTTCAGAATCTCGTC | GATGTATTCAGCATTGGCCACGATGCT |
| OR12 | CTGGCTGCCTTGGACTGTT | TTCCAAGTCTTCCCCAAGCT | CSP21-NGSP | CGCGTTGCTCTGGCTTGTCAAGTATA | GCGATGGAGACATCGACGTACACAACC |
| OR13 | AATGAGTTGACGCTACAAG | CTTATCATCATCACCAGCAA | CSP23-GSP | TCTGAGCCAGTTTTTTGCTTCGCA | AGTCAGAGCGCCTTTTGAAAGCATACG |
| OR14 | GCTCTTTACTGATCTGCCTTGC | AGTTGGTTATTTCTACGCTCGC | CSP23-NGSP | CTCATTTTCTAAGGCGTCGGGCAG | GCACTCCCGACGGAAAAGCCCTAA |
| OR15 | GAGTTCGGTCAATATCTGCTGC | CGGGGATTAGTACGGAACCA | OR2(OR83b)  -GSP | CCGAGCACCGAAGCAAACAAATCAA | GATTTGTTTGCTTCGGTGCTCGGT |
| OR16 | GCAAACTGGGTATACGCTGATT | CGAATATGCGGTGCGAAG |
